# “Rethinking High‐KDPI Kidneys: A Multidomain Approach to Predicting Success”

**DOI:** 10.1111/ctr.70472

**Published:** 2026-02-04

**Authors:** Xingyu Zhang, Chethan Puttarajappa, Frank Spitz, Jason Mial‐Anthony, Berkay Demirors, Abiha Abdullah, Vrishketan Sethi, Matthew Yu‐Sheng Lin, Andrew Crane, Winn Cashion, Charbel Elias, Han Shwe, Timothy Fokken, Bradley Phelp, Ryugen Takahashi, Hao Liu, Christof Kaltenmeier, Stalin Dharmayan, Vikraman Gunabushanam, Armando Ganoza, Martin N.Wijkstrom, Amit Tevar, Michele Molinari

**Affiliations:** ^1^ Biostatistics and Epidemiology, School of Health and Rehabilitation Sciences University of Pittsburgh Pittsburgh Pennsylvania USA; ^2^ Department of Medicine Division of Nephrology University of Pittsburgh Medical Center Pittsburgh Pennsylvania USA; ^3^ Department of Surgery, Division of Abdominal Transplantation University of Pittsburgh Medical Center Pittsburgh Pennsylvania USA; ^4^ Department of Surgery, School of Medicine, College of Medicine Kaohsiung Medical University Kaohsiung Taiwan; ^5^ Department of Surgery, Division of Abdominal Transplantation Willis Knighton J. C. McDonald Transplant Center Shreveport Louisiana USA; ^6^ Department of Medicine, Division of Nephrology Maine Health Portland Maine USA; ^7^ Department of Surgery, Division of Abdominal Transplantation Houston Methodist J.C. Walter Jr. Transplant Center Houston Texas USA; ^8^ Department of Surgery, Division of Abdominal Transplantation Medical University of South Carolina Charleston South Carolina USA

**Keywords:** high‐kdpi, kidney donor profile index (kdpi), kidney transplant, post‐transplant outcomes, risk prediction

## Abstract

**Background:**

The allocation and acceptance of deceased‐donor kidneys in the United States is influenced by theKidney Donor Profile Index (KDPI). We conducted a national analysis of high‐KDPI kidney transplants performed from 2014 to 2021 to identify key predictors of post‐transplant outcomes beyond those incorporated in KDPI.

**Methods:**

This retrospective cohort study used data extracted from the Scientific Registry of Transplant Recipients (SRTR). Adult, first‐time recipients of kidney‐only deceased‐donor transplants with KDPI greater than 85% were included. Regression models were used to identify independent predictors of delayed graft function (DGF), primary graft nonfunction (PGNF), patient survival, overall graft survival, and death‐censored graft survival.

**Results:**

Among 4,911 recipients, DGF occurred in 33.8% and PGNF in 4.0%. DGF was independently associated with donation after circulatory death, terminal donor creatinine > 1.5 mg/dL, recipient obesity, dialysis duration > 3 years, and cold ischemia time (CIT) ≥ 24 h, whereas machine perfusion was protective. PGNF was associated with donation after circulatory death, terminal donor creatinine > 2.0 mg/dL, high‐risk cytomegalovirus (CMV) serostatus, and donor injury patterns within the high‐KDPI range, including younger donor age. Five‐year patient and graft survival were 72% and 62%, respectively. Graft loss was independently associated with DGF, elevated intrarenal resistive index (RI), recipient diabetes, prolonged dialysis exposure, and high‐risk CMV/EBV serostatus.

**Conclusions:**

Outcomes after high‐KDPI kidney transplantation reflect both KDPI‐defined donor risk and additional recipient, immunologic, and perioperative factors. A multidomain, offer‐time assessment may support more individualized acceptance decisions and improve utilization.

## 1 Introduction

The Kidney Donor Profile Index (KDPI) was introduced in the United States (U.S.) in 2014 with implementation of the revised Kidney Allocation System (KAS) [1, 2, 3, 4, 5]. Derived from the Kidney Donor Risk Index (KDRI), KDPI integrates multiple donor characteristics—including age, body size, donation after circulatory death, history of hypertension or diabetes, terminal serum creatinine, hepatitis C virus status, and cause of death—into a percentile score relative to the contemporary U.S. donor pool [2]. KDPI is used to stratify donor kidney quality and has become a central metric guiding both organ allocation and clinical decisions regarding organ acceptance.

KDPI ranges from 0% to 100%, with higher values indicating kidneys associated with greater donor‐related risk and shorter expected graft survival [2]. However, KDPI is inherently donor‐centered and does not account for other important determinants of posttransplant outcomes such as recipient comorbidity burden, immunologic profile, and perioperative or logistical factors such as cold ischemic time and graft preservation technologies [2, 3, 4, 5]. Consequently, KDPI alone represents an important but incomplete predictive tool, and reliance on KDPI in isolation encourages risk‐averse decisions that contribute to the high rate of kidney nonutilization in the U.S [2, 3, 4, 5, 6].

Prior studies have demonstrated that carefully selected high‐KDPI kidneys can achieve acceptable outcomes, particularly among older recipients and candidates at high risk of dialysis‐related mortality [3, 6, 7, 8, 9, 10, 11]. Despite this evidence, reluctance to accept high‐KDPI organs persists, reflecting uncertainty regarding which donor–recipient combinations derive the greatest benefit from transplantation [12]. The lack of an integrated, offer‐time framework that consider donor risk, recipient vulnerability, immunologic profile, and perioperative conditions continues to limit consistent, evidence‐based acceptance decisions across transplant centers and providers [3, 6, 8, 9, 10, 11, 13, 14].

To address this gap, we performed an in‐depth analysis of kidney transplant recipients who received kidneys with KDPI > 85%. The objectives of this study were threefold: (1) to characterize short‐ and long‐term outcomes after transplantation of high‐KDPI kidneys in contemporary U.S. practice; (2) to identify independent donor, recipient, immunological and perioperative predictors of outcomes within recipient of high‐KDPI kidneys; and (3) to synthesize these predictors into a multidomain, evidence‐informed framework designed to complement KDPI and support individualized, offer‐time acceptance decisions.

## 2 Methods

### 2.1 Study Design and Inclusion and Exclusion Criteria

This retrospective national cohort study included adults (≥ 18 years) who underwent kidney transplantation with deceased‐donor grafts having KDPI > 85% between December 4, 2014 (implementation of the new KAS), and December 31, 2021. Patients were followed through December 31, 2024, allowing assessment of both early and long‐term outcomes. Eligibility was restricted to first‐time, kidney‐only transplant recipients. We excluded living‐donor transplants, en bloc or dual kidney transplants, multi‐organ transplants, and ABO‐incompatible grafts to maintain clinical homogeneity and limit confounding from nonstandard allocation pathways.

### 2.2 Data Source

Data were obtained from the Scientific Registry of Transplant Recipients (SRTR), a national database containing detailed information on all U.S. organ donors, waitlisted candidates, and transplant recipients. SRTR data are submitted by members of the Organ Procurement and Transplantation Network (OPTN) and are administratively and scientifically overseen by the Health Resources and Services Administration (HRSA), U.S. Department of Health and Human Services.

### 2.3 KDPI Calculation and Stratification

KDPI was derived using the year‐invariant KDRI based on the Rao formula, which incorporates donor demographics, comorbidities, laboratory values, modality of death, and hepatitis C virus status [2]. Annual UNOS conversion tables were used to translate KDRI into KDPI, thereby accounting for temporal changes in the national donor pool [15].

Because KDPI already incorporates several donor attributes evaluated in this study (e.g., donation after circulatory death and terminal creatinine), analyses were explicitly designed to distinguish (i) KDPI components that remain prognostically relevant within the high‐KDPI stratum from (ii) additional donor, recipient, immunologic, and perioperative domains that provide independent prognostic information beyond KDPI. Recipients were empirically categorized into KDPI strata of 86–90%, 91%–95%, and > 95% to assess whether increasing KDPI within the high‐risk range exhibited a dose‐dependent association with post‐transplant outcomes.

### 2.4 Variables

All donor, recipient, immunologic, and perioperative variables extracted from the SRTR, and included in the analyses are summarized in Supplementary Table S1.

### 2.5 Donor Variables

Donor characteristics were age, sex, race‐ethnicity, height, weight, hypertension, diabetes, cause of death, HCV status, donation type (donation after brain death vs. donation after circulatory death), terminal and peak creatinine, terminal sodium, Centers for Disease Control and Prevention (CDC) high‐risk status, use of vasopressors, and presence of proteinuria. When available, procurement biopsy data—percent glomerulosclerosis and biopsy performance status—were included.

### 2.6 Recipient Variables

Recipient variables were age, sex, race‐ethnicity, body mass index (BMI) (WHO categories), HLA mismatch, Epstein‐Barr virus (EBV) and cytomegalovirus (CMV) serostatus, functional status Karnofsky Performance Score (KPS) [16], comorbidities (diabetes, peripheral vascular disease), cPRA, cause of end‐stage renal disease (ESRD), dialysis duration, and KDPI category. Functional status was classified as good (KPS > 70%), fair (50%–70%), or poor (< 50%). EBV and CMV risk levels were defined by donor (D) and recipient (R) serostatus (low: D−/R−; intermediate: any R+; high: D+/R−).

### 2.7 Perioperative and Organ‐Specific Variables

Perioperative variables were cold ischemia time (CIT; categorized as 0–11.9, 12–17.9, 18–23.9, and ≥ 24 h), use of machine perfusion, and intrarenal resistive index (RI) measured during ex situ perfusion, categorized as ≤ 0.4 or > 0.4. The RI threshold of 0.4 was selected based on previous experimental and clinical studies demonstrating that resistance values at or above this level are associated with impaired microvascular perfusion and increased susceptibility to ischemia–reperfusion injury [17, 18]. Variables related to surgical complexity, including warm ischemia time and anastomotic duration, are not consistently captured in SRTR and were therefore excluded from the final analysis.

### 2.8 Outcomes

*The primary outcomes were:*

1. incidence of DGF, defined as the requirement for dialysis within the first 7 days after transplantation; and
2. incidence of PGNF, defined as irreversible failure of the kidney allograft to achieve meaningful function, resulting in permanent dialysis dependence or graft nephrectomy within 90 days after transplantation, in accordance with SRTR reporting standards.

*Secondary outcomes were:*

1. patient survival, defined as time from transplantation to all‐cause mortality.
2. overall graft survival, defined as time to graft failure, including return to dialysis, relisting, nephrectomy, or retransplantation; and
3. death‐censored graft survival, defined as time to graft failure with censoring at death with a functioning graft.

### 2.9 Statistical Analysis

Continuous variables were assessed for normality using the Shapiro‐Wilk test and summarized as mean (SD) or median (IQR), as appropriate. Group comparisons were performed using *t* tests, analysis of variance, or Kruskal‐Wallis tests for continuous variables, and χ^2^ tests for categorical variables, which were summarized as counts and percentages.

Time‐to‐event outcomes were analyzed using Kaplan‐Meier methods and compared with the log‐rank test, with censoring at death with a functioning graft or at last follow‐up, as appropriate.

Multivariable regression models were used to identify independent predictors of post‐transplant outcomes. Logistic regression was used to estimate adjusted odds ratios (aORs) for DGF and PGNF. Cox proportional hazards regression was used to estimate adjusted hazard ratios (aHRs) for patient survival, overall graft survival, and death‐censored graft survival. Proportional hazards assumptions were assessed using Schoenfeld residuals.

Sensitivity analyses were prespecified to assess robustness. Models were refitted after excluding preemptively transplanted recipients to address potential misclassification of PGNF. In addition, Fine‐Gray subdistribution hazard models were performed as sensitivity analyses for death‐censored graft survival, treating death as a competing event rather than a censoring event, consistent with reviewer recommendations.

All covariates described in the Methods were considered for model entry and were selected a priori based on biological plausibility, clinical relevance, and prior evidence. Each model was initially fitted with a full covariate set, followed by backward elimination using a retention threshold of *P* ≤.10. Donor age and donation type, key components of KDPI, were retained in all models regardless of statistical significance. CIT was included in all models to evaluate their direct associations with outcomes, and survival analyses were additionally adjusted for the occurrence of DGF (Supplementary Table S2).

Multicollinearity was assessed using variance inflation factors and addressed through variable modification or exclusion as appropriate. Bonferroni correction was applied within outcome categories to account for multiple comparisons. Statistical significance was defined as a 2‐sided *p* < 0.05. All analyses were performed using SAS version 9.4 (SAS Institute Inc).

### 2.10 Integrated Risk Pyramid Creation

Independent predictors identified in fully adjusted models were synthesized into an Integrated Risk Pyramid, organizing risk factors into donor‐related, recipient‐related, immunologic, and technical‐logistic domains. To align with real‐world acceptance decisions, predictors were further categorized by modifiability at the time of organ offer. Potentially modifiable factors included CIT, use of machine perfusion, and recipient selection emphasizing physiological reserve and shorter dialysis duration.

This framework was intentionally designed as an evidence‐informed, offer‐time decision support tool rather than a post hoc risk score, complementing KDPI by integrating additional domains that influence outcomes within the high‐KDPI population.

### 2.11 Ethics Approval and Compliance

The Institutional Review Board approved this study (PRO13060220) and waived informed consent due to the use of de‐identified SRTR data. Research was conducted according to the Declaration of Helsinki [19] and the Declaration of Istanbul [20]. Reporting followed Strengthening the Reporting of Observational Studies in Epidemiology (STROBE) guidelines [21].

## 3 Results

### 3.1 Study Population

The study included 4,911 kidney transplant recipients (Table 1), of whom 46.5% received kidneys with KDPI 86–90 (*n* = 2,334), 33.9% with KDPI 91–95 (*n* = 1,664), and 18.6% with KDPI > 95 (*n* = 913). The median recipient age was 65 years (IQR, 59–70), and 63.5% were male. The cohort was racially and ethnically diverse, comprising 35.3% White, 35.1% Black, and 19.6% Hispanic/Latino recipients. Metabolic comorbidity was common, with 53.7% of recipients having diabetes, which was also the leading cause of end‐stage kidney disease (47.8%), followed by hypertension (28.2%). Functional status was generally preserved, with 48.0% of recipients having a KPS > 70, and sensitization was low, with a median calculated panel reactive antibody (cPRA) of 0% and only 1.8% classified as highly sensitized (cPRA ≥ 95%). The extent of missing data for recipient‐, donor‐, immunologic‐, and perioperative variables analyzed in this study is summarized in Supplementary Table S3.

**TABLE 1:** Baseline characteristics of recipients of kidneys with KDPI >85%, stratified by KDPI category. Continuous variables are shown as median (IQR) and categorical variables as counts and percentages. *P*‐values reflect comparisons across KDPI groups.

Characteristics	All recipients (*n* = 4911)	KDPI 86–90 (*n* = 2334)	KDPI 91–95 (*n* = 1664)	KDPI >95 (*n* = 913)	*p* value
**Sex, n. (%)**					
Female	1792 (36.5)	855 (36.6)	601 (36.1)	336 (36.8)	0.924
Male	3119 (63.5)	1479 (63.4)	1063 (63.9)	577 (63.2)	
**Age, years, median (IQR)**	65.0 (59.0–70.0)	64.0 (57.0–69.0)	65.0 (59.0–70.0)	67.0 (62.0–71.0)	< 0.001
< 35	16 (0.3)	9 (0.4)	6 (0.4)	1 (0.1)	< 0.001
35–49	253 (5.2)	156 (6.7)	81 (4.9)	16 (1.8)	
50–65	2110 (43.0)	1100 (47.1)	699 (42.0)	311 (34.1)	
> 65	2532 (51.6)	1069 (45.8)	878 (52.8)	585 (64.1)	
**Race‐ethnicity, n. (%)**					
White‐Caucasian	1736 (35.3)	822 (35.2)	580 (34.9)	334 (36.6)	0.427
Black‐African American	1722 (35.1)	797 (34.1)	609 (36.6)	316 (34.6)	
Hispanic‐Latino	961 (19.6)	483 (20.7)	316 (19.0)	162 (17.7)	
Asian	424 (8.6)	196 (8.4)	139 (8.4)	89 (9.7)	
Other	68 (1.4)	36 (1.5)	20 (1.2)	12 (1.3)	
**Etiology of renal failure**					
Diabetic nephropathy	2189 (47.8)	1047 (47.9)	704 (45.6)	438 (51.7)	0.002
Hypertension	1290 (28.2)	590 (27.0)	461 (29.9)	239 (28.2)	
Glomerulonephritis	550 (12.0)	277 (12.7)	203 (13.2)	70 (8.3)	
Other	549 (12.0)	274 (12.5)	175 (11.3)	100 (11.8)	
**Body Mass Index (BMI), median (IQR)**	28.2 (24.8–31.9)	28.5 (25.1–32.3)	28.0 (24.5–31.7)	27.5 (24.5–31.4)	0.081
< 18.5	149 (3.0)	72 (3.1)	56 (3.4)	21 (2.3)	0.027
18.5–24.9	1226 (25.0)	537 (23.0)	433 (26.0)	256 (28.0)	
25–29.9	1779 (36.2)	851 (36.5)	594 (35.7)	334 (36.6)	
≥ 30	1757 (35.8)	874 (37.4)	581 (34.9)	302 (33.1)	
**Patients on dialysis, n. (%)**	4530 (92.2)	2167 (92.8)	1535 (92.2)	828 (90.7)	0.336
**Preemptive transplantation, n (%)**	381 (7.8)	167 (7.2)	129 (7.8)	85 (9.3)	0.128
**Dialysis duration, years, (IQR)**	4.16 (2.28–6.22)	4.35 (2.43–6.50)	4.18 (2.40–6.16)	3.54 (1.79–5.53)	< 0.001
0–3	1547(31.5)	693 (29.7)	514 (30.9)	340 (37.2)	< 0.001
3.1–5	1249 (25.4)	568 (24.3)	446 (26.8)	235 (25.7)	
> 5	1734 (35.3)	906 (38.8)	575 (34.6)	253 (27.7)	
**History of diabetes**	2638 (53.7)	1253 (53.7)	868 (52.2)	517 (56.6)	0.094
**History of Peripheral vascular disease**	730 (14.9)	351 (15.0)	244 (14.7)	135 (14.8)	0.944
**Karnofsky Functional Index**					
< 50	108 (2.3)	63 (2.8)	29 (1.8)	16 (1.8)	0.056
50–70	2375 (49.7)	1123 (49.6)	834 (51.3)	418 (47.2)	
> 70	2292 (48.0)	1079 (47.6)	762 (46.9)	451 (51.0)	
**cPRA (%)**					
0–84	4780 (97.4)	2258 (96.7)	1628 (97.9)	894 (97.9)	0.023
85–90	17 (0.3)	14 (0.6)	2 (0.1)	1 (0.1)	
90–94	24 (0.5)	16 (0.7)	3 (0.2)	5 (0.5)	
≥ 95	89 (1.8)	46 (2.0)	30 (1.8)	13 (1.4)	
**EBV serostatus**					
Low risk	9 (0.2)	7 (0.3)	2 (0.1)	0 (0.0)	0.368
Intermediate risk	4333 (94.3)	2038 (94.2)	1481 (94.5)	814 (94.0)	
High risk	255 (5.5)	118 (5.5)	85 (5.4)	52 (6.0)	
**CMV status**					
Low risk	417 (8.6)	207 (9.0)	150 (9.2)	60 (6.7)	0.214
Intermediate risk	3434 (71.0)	1619 (70.5)	1164 (71.0)	651 (72.2)	
High risk	987 (20.4)	471 (20.5)	325 (19.8)	191 (21.2)	
**Transplant center volume**					
≤ 100	3597 (73.2)	1808 (77.5)	1197 (71.9)	592 (64.8)	< 0.001
101–200	1083 (22.1)	433 (18.6)	381 (22.9)	269 (29.5)	
> 200	231 (4.7)	93 (4.0)	86 (5.2)	52 (5.7)	
**HLA mismatch**					
0–3	652 (13.3)	334 (14.3)	210 (12.6)	108 (11.8)	0.063
4	1192 (24.3)	563 (24.1)	411 (24.7)	218 (23.9)	
5	1855 (37.8)	905 (38.8)	609 (36.6)	341 (37.3)	
6	1212 (24.7)	532 (22.8)	434 (26.1)	246 (26.9)	

**Abbreviations**: c‐PRA, calculated panel reactive antibody; CMV, cytomegalovirus; EBV, Epstein‐Barr virus.

Donors had a median age of 62 years (IQR, 57–66), and 54.0% were female (Table 2). Most kidneys were recovered from donation after brain death (91.5%), with 8.6% from donation after circulatory death. Donor causes of death reflected contemporary national trends, with cerebrovascular accident accounting for 67.1%, followed by anoxia (22.0%) and head trauma (9.9%). Donor hypertension (79.6%) and diabetes (28.7%) were common, and 11.7% met CDC high‐risk criteria.

**TABLE 2:** Donor characteristics for kidneys with KDPI >85%, stratified by KDPI category. Continuous variables are shown as median (IQR) and categorical variables as counts and percentages. *P*‐values reflect comparisons across KDPI groups.

Characteristics	All donors (*n*.4,911)	KDPI 86–90 (*n*.2,334)	KDPI91–95 (*n*.1,664)	KDPI > 95 (*n*.913)	*P* values
**Sex, n. (%)**					
Female	2650 (54.0)	1190 (51.0)	936 (56.3)	524 (57.4)	< 0.001
Male	2261 (46.0)	1144 (49.0)	728 (43.7)	389 (42.6)	
**Age, years, median (IQR)**	62.0 (57.0–66.0)	60.0 (56.0–64.0)	62.0 (58.0–66.0)	65.0 (61.0–69.0)	< 0.001
Age < 35 yrs	230 (5.0)	150 (6.0)	80 (5.0)	0 (0.0)	< 0.001
Age 35–49 yrs	394 (8.0)	141 (6.0)	52 (3.0)	1 (0.1)	
Age 50–64 yrs	3157 (64.3)	1674 (71.7)	1059 (63.6)	424 (46.4)	
Age > 65 yrs	1537 (31.3)	504 (21.6)	545 (32.8)	488 (53.5)	
**Race‐ethnicity, n. (%)**					
White‐Caucasian	2426 (49.4)	1327 (56.9)	774 (46.5)	325 (35.6)	< 0.001
Black African American	1520 (31.0)	575 (24.6)	551 (33.1)	394 (43.2)	
Hispanic‐Latino	724 (14.7)	334 (14.3)	248 (14.9)	142 (15.6)	
Asian	218 (4.4)	84 (3.6)	85 (5.1)	49 (5.4)	
Other	23 (0.5)	14 (0.6)	6 (3.6)	3 (0.3)	
**Death type, n. (%)**					
Brain death (DBD)	4491 (91.5)	2069 (88.6)	1544 (92.8)	878 (96.2)	< 0.001
Cardiocirculatory arrest (DCD)	420 (8.6)	265 (11.4)	120 (7.2)	35 (3.8)	
**Body Mass Index, median (IQR)**	28.0 (24.2–32.9)	28.4 (24.2–33.1)	27.8 (24.2–32.9)	27.4 (24.0–32.4)	0.001
< 18.5	138 (2.7)	69 (3.0)	44 (2.6)	20 (2.2)	0.016
18.5–24.9	1388 (27.7)	605 (25.9)	466 (28.0)	277 (30.3)	
25–29.9	1580 (32.2)	740 (31.7)	525 (31.6)	315 (34.5)	
≥ 30	1850 (37.7)	920 (39.4)	629 (37.8)	301 (33.0)	
**Primary cause of death, n. (%)**					
Anoxia	1078 (22.0)	585 (25.1)	346 (20.8)	147 (16.1)	< 0.001
Cerebrovascular/stroke	3297 (67.1)	1486 (62.7)	1158 (69.6)	681 (74.6)	
Head trauma	486 (9.9)	267 (11.4)	143 (8.6)	76 (8.3)	
CNS tumor	72 (1.0)	8 (0.3)	20 (1.0)	6 (0.2)	
Unknown/Other	380 (8.0)	160 (7.0)	150 (9.0)	70 (8.0)	
**History of diabetes, n. (%)**	1384 (28.7)	585 (25.4)	494 (30.2)	359 (38.9)	< 0.001
**History of HCV, n. (%)**	597 (12.2)	232 (10.0)	191 (11.5)	174 (19.1)	< 0.001
**History of hypertension, n. (%)**	3869 (79.6)	1712 (74.4)	1581 (95.0)	818 (89.7)	< 0.001
**Proteinuria, n, (%)**	2329 (48.7)	1139 (48.4)	805 (48.4)	448 (49.1)	< 0.001
**Peak serum creatinine, median (IQR)**	1.3 (1.0–1.8)	1.4 (1.1–1.8)	1.3 (1.0–1.8)	1.3 (1.1–1.7)	0.016
< 1.0 (mg/dL)	964 (19.6)	454 (19.5)	351 (21.1)	159 (17.4)	0.937
1.0–1.49 (mg/dL)	2151 (43.8)	1003 (43.0)	705 (42.4)	443 (48.5)	0.016
1.5–2.0 (mg/dL)	1002 (20.4)	479 (20.5)	336 (20.2)	189 (20.7)	
> 2.0 (mg/dL)	792 (16.1)	398 (17.1)	277 (16.4)	122 (13.4)	
**Terminal sodium, mEq/L, median (IQR)**	149.0 (144.0–154.0)	149.0 (144.0–154.0)	149.0 (144.0–154.0)	149.0 (144.0–154.0)	0.991
< 135	116 (2.4)	59 (2.5)	37 (2.2)	20 (2.2)	0.882
135–145	1436 (29.2)	692 (29.6)	488 (29.3)	256 (28.0)	
> 145	3359 (68.4)	1587 (68.0)	1139 (68.4)	653 (69.3)	
**CDC high risk, n. (%)**	577 (11.7)	305 (13.1)	183 (11.0)	89 (9.7)	0.014
**Use of Vasopressor, n. (%)**	2471 (50.3)	1126 (48.2)	872 (52.4)	473 (51.8)	0.015

**Abbreviations**: CDC, center for disease control and prevention; HCV, hepatitis C virus.

Assessment of organ quality included preimplantation biopsy in 94.9% of grafts, with 58.2% demonstrating ≤ 5% glomerulosclerosis and fewer than 10% showing > 15% glomerulosclerosis. Machine perfusion was used in 46.9% of cases, and among pumped kidneys with available data, 96.7% had intrarenal RI values ≤ 0.4. CIT was similar across KDPI strata, with a median of 19.3 h (IQR, 14.5–24.9) and comparable distributions across prespecified ischemia intervals. Detailed organ‐ and logistics‐related characteristics are presented in Table 3.

**TABLE 3:** Organ characteristics and duration of cold ischemia across KDPI categories.

Organ characteristics and cold ischemia time	All organs (*n*. 4911)	KDPI 86%–90% (*n*. 2334)	KDPI 91%–95% (*n*. 1664)	KDPI > 95% (*n*. 913)	*p* Value
**Preoperative biopsy, n. (%)**					< 0.001
Not obtained	252 (5.1)	162 (6.9)	70 (4.2)	20 (2.2)	
Obtained	4659 (94.9)	2172 (93.1)	1594 (95.8)	893 (97.8)	
**Percentage of glomerulosclerosis, n. (%)**					
0%–5%	2711 (58.5)	1275 (59.0)	915 (57.7)	521 (58.5)	0.294
6%–10%	1068 (23.0)	501 (23.2)	343 (21.6)	224 (25.2)	
11%–15%	440 (9.5)	202 (9.3)	166 (10.5)	72 (8.1)	
16%–20%	237 (5.1)	103 (4.8)	95 (6.0)	39 (4.4)	
> 20%	174 (3.8)	76 (3.5)	65 (4.1)	33 (3.7)	
Indeterminate	7 (0.2)	4 (0.2)	2 (0.1)	1 (0.1)	
**Kidneys put on perfusion pump, n. (%)**	2307 (46.9)	1122 (48.0)	743 (44.6)	442 (48.4)	0.054
**Resistive index**					0.097
≤ 0.4	2190 (44.6)	1065 (45.6)	711 (42.7)	414 (45.3)	
> 0.4	117 (2.4)	57 (2.4)	32 (1.9)	28 (3.1)	
Not available	2604 (53.0)	1212 (46.5)	921 (35.4)	471 (51.6)	
**Cold ischemia time, hours, median (IQR)**	19.3 (14.5–24.9)	19.3 (14.4–25.0)	19.1 (14.6–24.7)	19.7 (15.0–24.8)	0.557
< 12	729 (15.1)	357 (15.6)	251 (15.3)	121 (13.4)	0.638
12–17.9	1301 (26.9)	597 (26.0)	457 (27.8)	247 (27.4)	
18–23.9	1438 (29.7)	693 (30.2)	471 (28.7)	274 (30.4)	
≥ 24	1366 (28.3)	646 (28.2)	462 (28.2)	258 (28.7)	

*Note:* RI (Resistive Index) available only for machine‐perfused grafts; ‘Not available’ reflects kidneys not on machine‐perfusion pumps.

### 3.2 Primary Outcomes

#### 3.2.1 DGF

**FIGURE 1:**
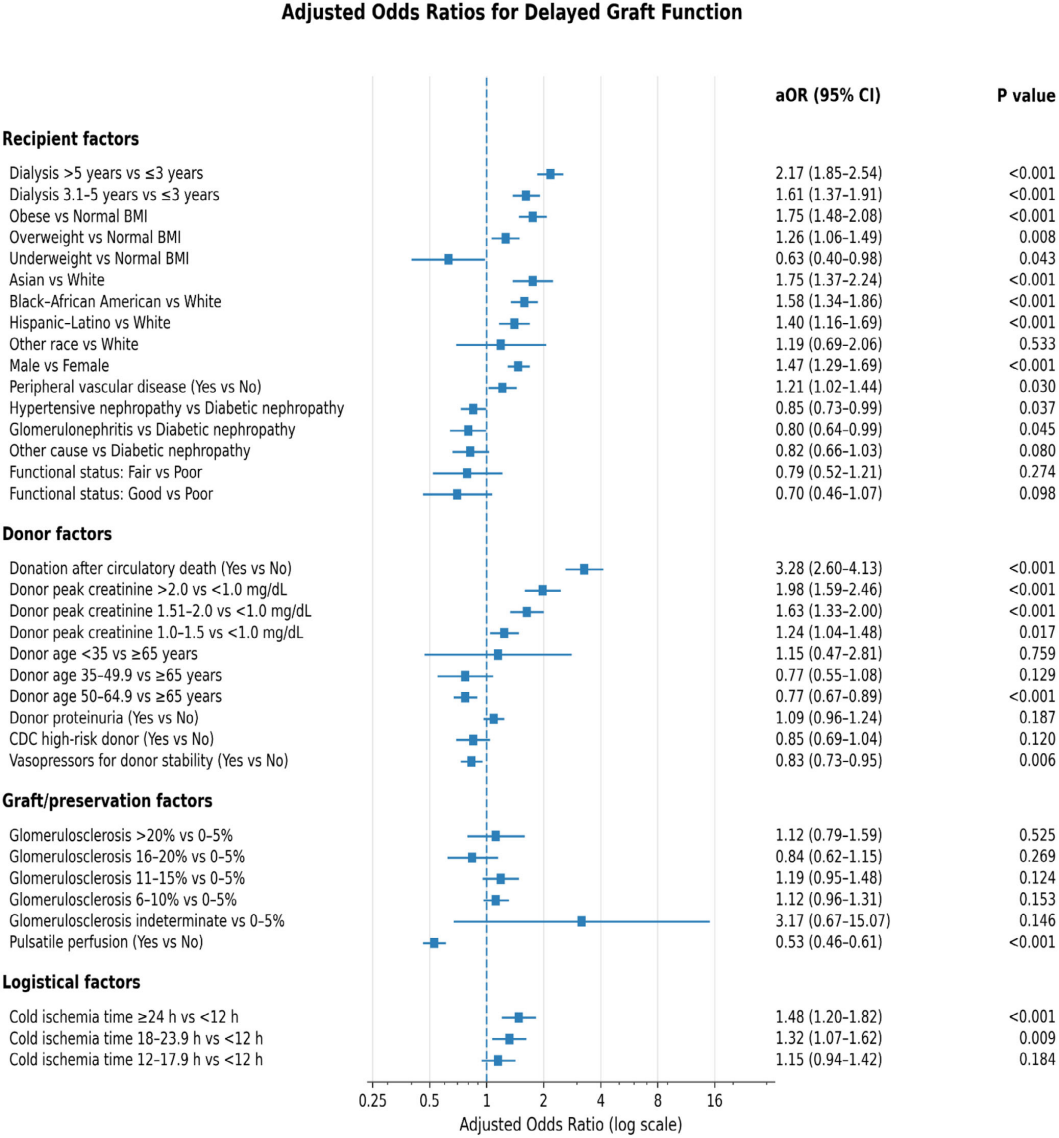
Multivariable predictors of delayed graft function. Adjusted odds ratios (aORs) for the risk of dialysis requirement within the first 7 days after transplantation, derived from the final multivariable logistic regression model adjusting for donor, recipient, immunologic, and technical‐logistic factors.

DGF occurred in 1,661 recipients (33.8%) and did not differ significantly across KDPI strata (35.1% for KDPI 86%–90%, 32.6% for KDPI 91%–95%, and 32.9% for KDPI > 95%; *p* = 0.20). Fully adjusted predictors of DGF are presented in Figure 1.

Among donor‐related factors, donation after circulatory death was the strongest predictor of DGF (adjusted odds ratio [aOR], 3.28; 95% CI, 2.60–4.13; *p* < 0.001). Elevated terminal donor serum creatinine was also independently associated with increased DGF risk, with a graded effect observed for levels of 1.51–2.0 mg/dL (aOR, 1.63; 95% CI, 1.33–2.00; *p* < 0.001) and greater than 2.0 mg/dL (aOR, 1.98; 95% CI, 1.59–2.47; *p* < 0.001).

Recipient‐level predictors included male sex (aOR, 1.49; 95% CI, 1.30–1.71; *p* < 0.001), increasing dialysis duration (3.1–5 years: aOR, 1.63; 95% CI, 1.38–1.92; *p* < 0.001; > 5 years: aOR, 2.20; 95% CI, 1.87–2.58; *p* < 0.001), obesity (BMI ≥ 30; aOR, 1.75; 95% CI, 1.47–2.08; *p* < 0.001), and peripheral vascular disease (aOR, 1.20; 95% CI, 1.01–1.44; *p* = 0.04). Prolonged CIT (≥ 24 h) was also independently associated with higher DGF risk (aOR, 1.47; 95% CI, 1.19–1.81; *p* < 0.001).

In contrast, donor vasopressor use prior to procurement was associated with lower odds of DGF (aOR, 0.83; 95% CI, 0.73–0.95; *p* = 0.006), as was preservation with pulsatile machine perfusion (aOR, 0.65; 95% CI, 0.48–0.89; *p* = 0.006).

Complete univariate analyses are provided in Supplementary Table S4.

#### 3.2.2 PGNF

**FIGURE 2:**
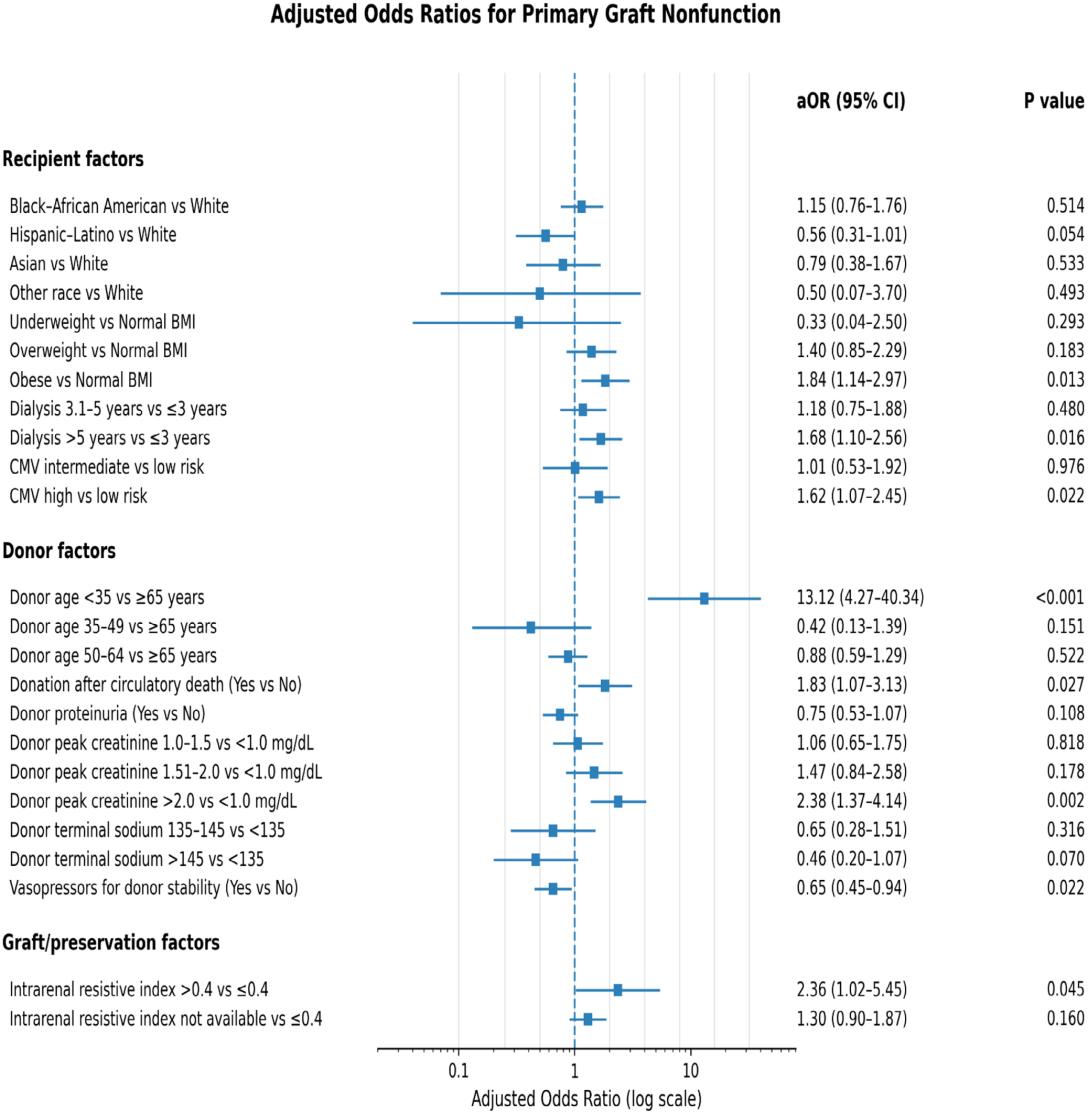
Multivariable predictors of primary graft nonfunction. Adjusted odds ratios (aORs) for the risk of primary graft nonfunction, derived from the final multivariable logistic regression model adjusting for donor, recipient, immunologic, and technical‐logistic factors.

PGNF occurred in 196 recipients (4.0%) and did not differ significantly across KDPI strata (4.0% for KDPI 86%–90%, 4.1% for KDPI 91%–95%, and 3.6% for KDPI > 95%; *p* = 0.79). Fully adjusted predictors of PGNF are shown in Figure 2.

Among donor‐related factors, donor age younger than 35 years was strongly associated with PGNF (adjusted odds ratio [aOR], 13.12; 95% CI, 4.27–40.34; *p* < 0.001). Additional donor and organ‐level predictors included terminal donor serum creatinine greater than 2.0 mg/dL (aOR, 2.38; 95% CI, 1.37–4.14; *p* = 0.002), elevated intrarenal RI during machine perfusion (RI > 0.4; aOR, 2.36; 95% CI, 1.02–5.45; *p* = 0.04), and donation after circulatory death (aOR, 1.83; 95% CI, 1.07–3.13; *p* = 0.03).

Recipient‐level factors independently associated with PGNF included obesity (BMI ≥ 30; aOR, 1.84; 95% CI, 1.14–2.97; *p* = 0.01), prolonged dialysis duration (> 5 years; aOR, 1.68; 95% CI, 1.10–2.56; *p* = 0.02), and high‐risk CMV serologic mismatch (donor positive/recipient negative; aOR, 1.62; 95% CI, 1.07–2.45; *p* = 0.02).

In contrast, donor vasopressor use prior to procurement was associated with a lower risk of PGNF (aOR, 0.65; 95% CI, 0.45–0.94; *p* = 0.02).

Complete univariate analyses are provided in Supplementary Table S5.

### 3.3 Secondary Outcomes

#### 3.3.1 Patient Survival

**FIGURE 3:**
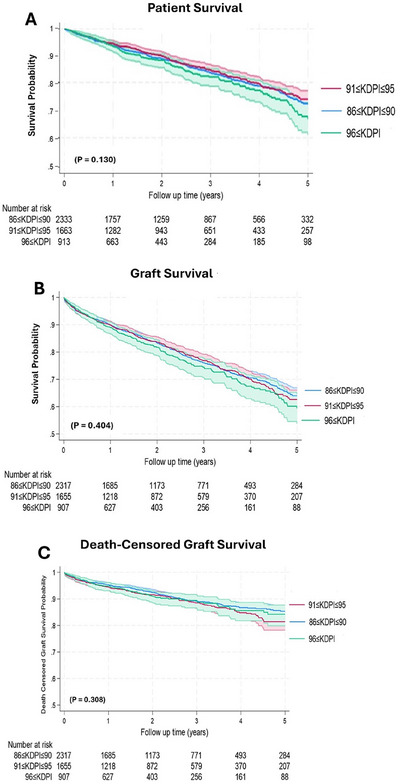
Patient and graft survival across high‐KDPI strata. (A) Patient survival following transplantation with kidneys of KDPI 86%–90%, 91%–95%, and >95%. (B) Overall graft survival, with graft failure defined as return to dialysis, relisting, nephrectomy, or retransplantation. (C) Death‐censored graft survival, with death occurring with a functioning graft treated as a censoring event. All survival curves were estimated using Kaplan‐Meier methods and compared using the log‐rank test.

The mean follow‐up duration was 5.47 years (95% CI, 5.39–5.55), during which 807 recipients died. Patient survival at 1, 3, and 5 years was 93%, 83%, and 72%, respectively, with no significant differences across KDPI strata (Figure 3A; *p* = 0.13).

**FIGURE 4:**
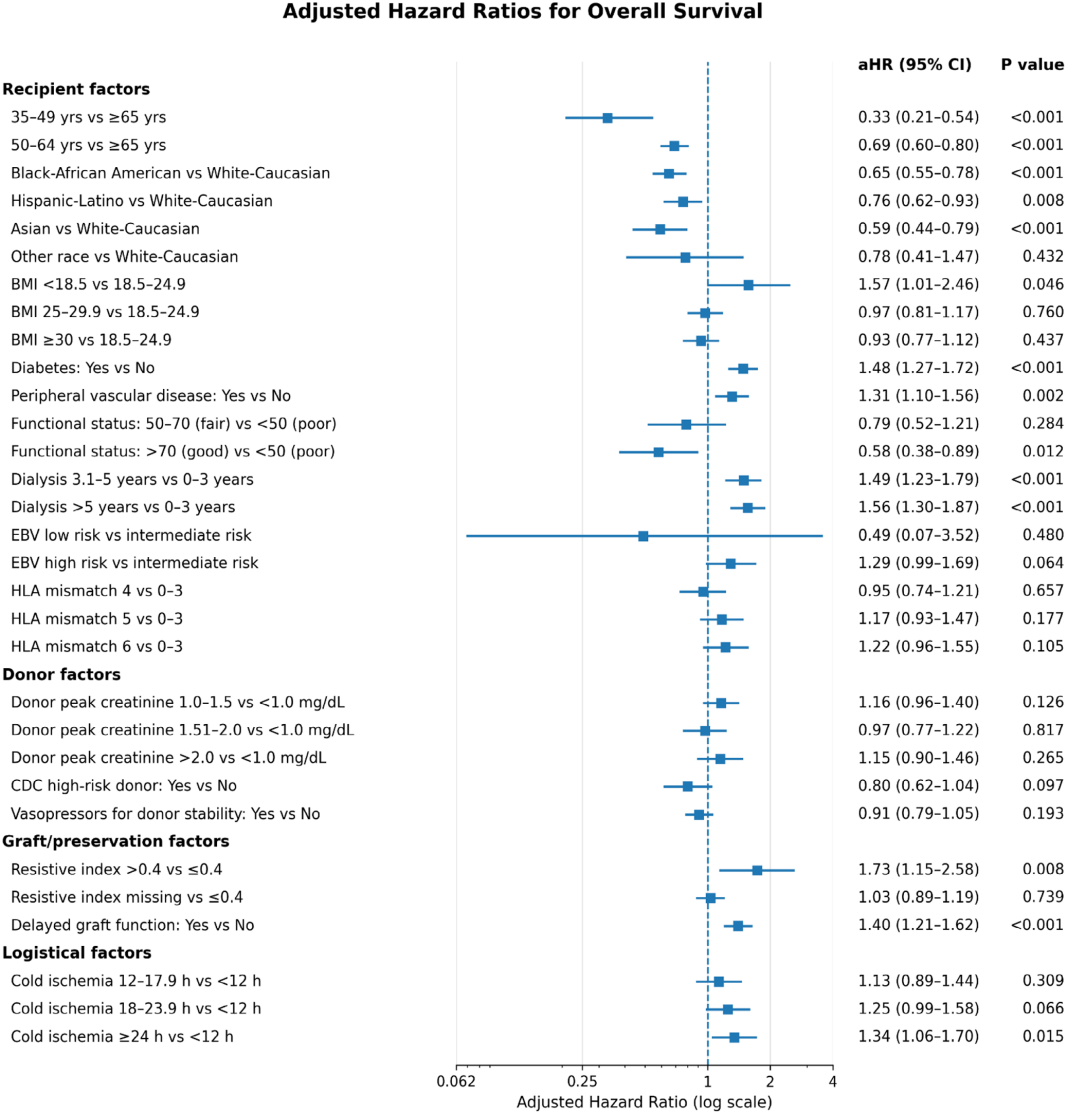
Multivariable predictors of recipient survival. Adjusted hazard ratios (aHRs) for all‐cause mortality derived from the final multivariable Cox proportional hazards regression model adjusting for donor, recipient, immunologic, and technical‐logistic factors.

In multivariable Cox regression analyses (Figure 4), dialysis duration emerged as the strongest predictor of mortality. Compared with recipients with less than 3 years of dialysis exposure, the risk of death increased among those with 3.1–5 years of dialysis (adjusted hazard ratio [aHR], 1.49; 95% CI, 1.23–1.79; *p* < 0.001) and those with more than 5 years (aHR, 1.56; 95% CI, 1.30–1.87; *p* < 0.001).

Additional recipient‐level factors independently associated with higher mortality included diabetes mellitus (aHR, 1.48; 95% CI, 1.27–1.72; *p* < 0.001), peripheral vascular disease (aHR, 1.31; 95% CI, 1.10–1.56; *p* = 0.002), and underweight status (BMI < 18.5; aHR, 1.57; 95% CI, 1.01–2.46; *p* = 0.046). Perioperative and organ‐related factors associated with increased mortality included DGF (aHR, 1.40; 95% CI, 1.21–1.62; *p* < 0.001), prolonged CIT (≥ 24 h; aHR, 1.34; 95% CI, 1.06–1.70; *p* = 0.02), and elevated intrarenal RI during machine perfusion (RI > 0.4; aHR, 1.73; 95% CI, 1.15–2.58; *p* = 0.007).

Complete univariate analyses are presented in Supplementary Table S6.

#### 3.3.2 Graft Survival

Overall graft survival at 1, 3, and 5 years was 89%, 75%, and 62%, respectively, with no significant differences acrossKDPI strata (Figure **3B**; *p* = 0.40).

**FIGURE 5:**
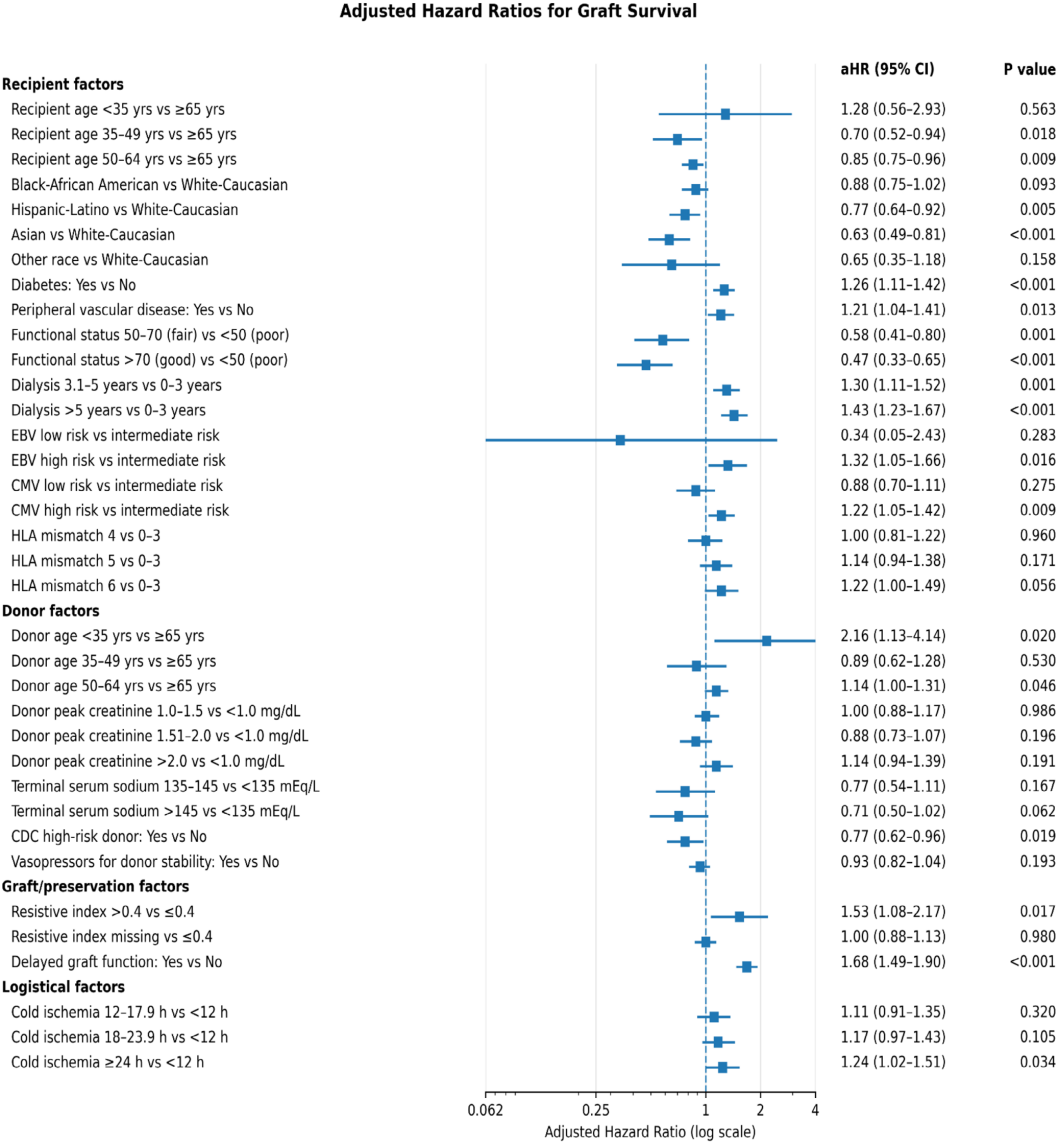
Multivariable predictors of graft loss. Adjusted hazard ratios (aHRs) for overall graft failure derived from the final multivariable Cox proportional hazards regression model adjusting for donor, recipient, immunologic, and technical‐logistic factors.

In the fully adjusted Cox regression model (Figure 5), DGF was the strongest predictor of graft loss (aHR, 1.68; 95% CI, 1.49–1.90; *p* < 0.001). Prolonged dialysis exposure was also independently associated with graft failure, with a graded increase in risk beginning at 3.1–5 years and reaching highest levels among recipients with dialysis duration exceeding 5 years (aHR, 1.43; 95% CI, 1.23–1.67; *p* < 0.001).

Among organ‐ and preservation‐related factors, elevated intrarenal RI during machine perfusion (RI > 0.4) was independently associated with graft loss (aHR, 1.53; 95% CI, 1.08–2.17; *p* = 0.02). Additional recipient and perioperative predictors included diabetes mellitus (aHR, 1.26; 95% CI, 1.11–1.42; *p* < 0.001), high‐risk Epstein–Barr virus serologic mismatch (donor positive/recipient negative; aHR, 1.32; 95% CI, 1.05–1.66; *p* = 0.02), and prolonged CIT (≥ 24 h; aHR, 1.24; 95% CI, 1.02–1.51; *p* = 0.03).

Complete univariate analyses are provided in Supplementary Table S7.

#### 3.3.3 Death Censored Graft Survival

Death‐censored graft survival at 1, 3, and 5 years was 96%, 90%, and 84%, respectively, with no significant differences across KDPI strata (Figure 3C; *p* = 0.31).

**FIGURE 6:**
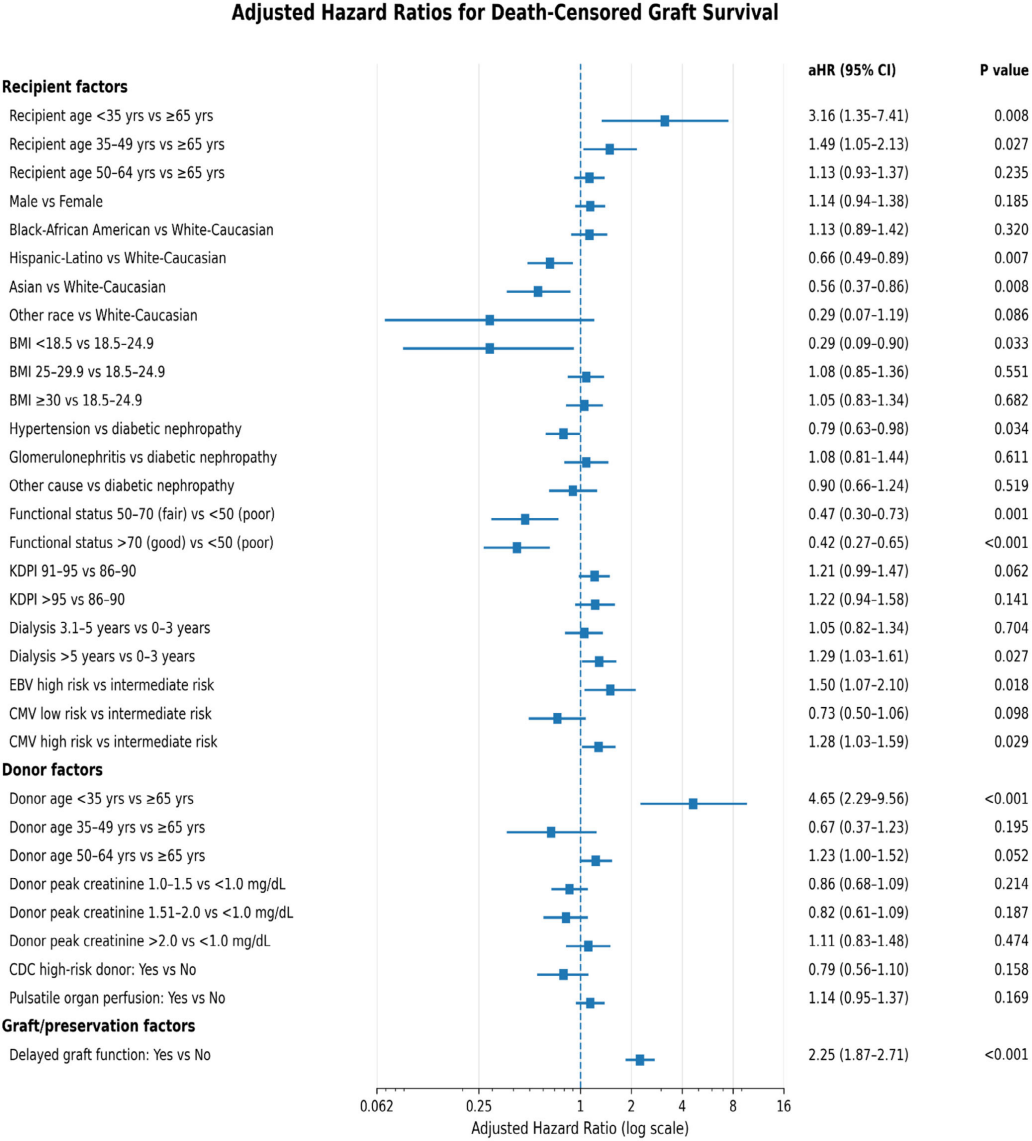
Multivariable predictors of death‐censored graft loss. Adjusted hazard ratios (aHRs) for death‐censored graft failure, with deaths occurring with a functioning graft treated as censoring events, derived from the final multivariable Cox proportional hazards regression model adjusting for donor, recipient, immunologic, and technical‐logistic factors.

In the fully adjusted Cox regression model (Figure 6), donor age younger than 35 years was the strongest predictor of death‐censored graft loss (aHR, 4.65; 95% CI, 2.26–9.56; *p* < 0.001). DGF conferred more than a twofold increase in risk (aHR, 2.25; 95% CI, 1.87–2.71; *p* < 0.001), and prolonged dialysis exposure greater than 5 years remained independently associated with graft failure (aHR, 1.29; 95% CI, 1.03–1.61; *p* = 0.03).

Immunological risk factors were also associated with adverse outcomes. High‐risk Epstein‐Barr virus serologic mismatch (donor positive/recipient negative) was associated with increased death‐censored graft loss (aHR, 1.50; 95% CI, 1.07–2.10; *p* = 0.02), as was high‐risk CMV serostatus (aHR, 1.28; 95% CI, 1.03–1.59; *p* = 0.03). Among recipient characteristics, recipient age younger than 35 years was independently associated with a higher risk of death‐censored graft loss (aHR, 3.16; 95% CI, 1.35–7.41; *p* = 0.008).

Complete univariate analyses are provided in Supplementary Table S8.

### 3.4 Sensitivity Analyses

#### 3.4.1 DGF

To address potential misclassification of DGF among preemptively transplanted recipients, multivariable models were refitted after excluding these patients (Supplementary Table S9). Exclusion of preemptive transplants did not meaningfully alter the direction, magnitude, or statistical significance of identified risk factors, supporting the robustness of the primary findings.

#### 3.4.2 PGNF

To similarly evaluate the robustness of findings for PGNF, analyses were repeated after exclusion of preemptively transplanted recipients (Supplementary Table S10). Results were consistent with the primary analysis, with no material changes in the direction or significance of associated risk factors.

#### 3.4.3 Competing‐Risk Graft Failure

To account for death as a competing event, Fine‐Gray sub distribution hazard models were performed (Supplementary Table S11). Associations were directionally consistent and comparable in magnitude to those observed in Cox death‐censored analyses, indicating that accounting for competing mortality did not materially alter identification of graft failure risk factors.

#### 3.4.4 Integrated Risk Pyramid

**FIGURE 7:**
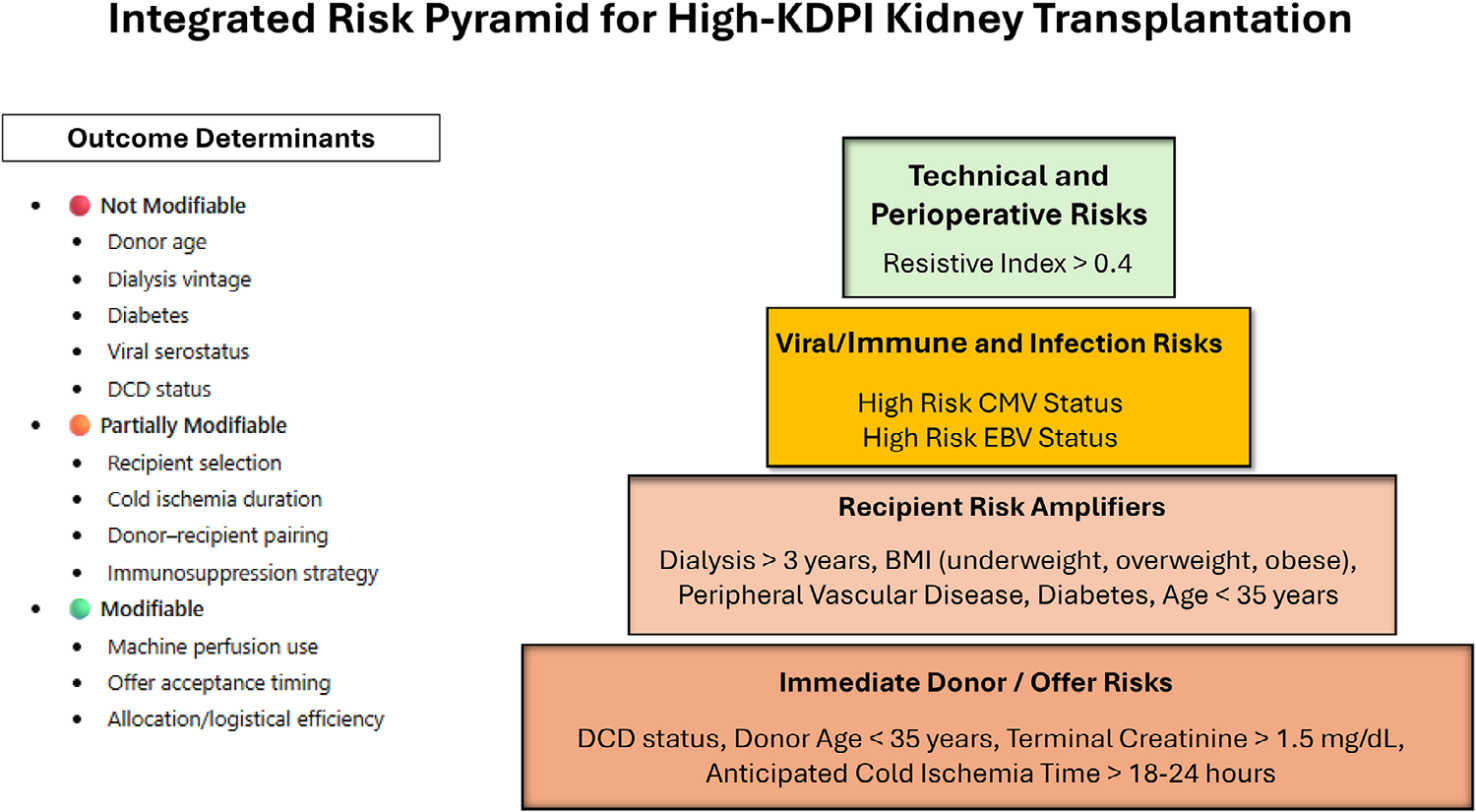
Integrated Risk Pyramid for high‐KDPI kidney transplantation. Independent predictors of adverse post‐transplant outcomes are organized into four interconnected domains: [1] donor and offer condition, reflecting organ quality and peri‐retrieval factors [2]; recipient vulnerability, capturing clinical characteristics associated with increased risk [3]; viral and immunologic susceptibility; and [4] technical and logistic execution, including ischemia exposure, perfusion parameters, and perioperative factors. Together, these domains form a structured, offer‐time framework illustrating how combined risks influence outcomes following high‐KDPI kidney transplantation.

Independent predictors identified in the fully adjusted models were synthesized into a multidomain risk pyramid (Table 4, Figure 7). This framework illustrates the interplay among four domains—donor and offer characteristics, recipient vulnerability, viral‐immunologic risk, and technical‐perioperative factors—that collectively influence outcomes after high‐KDPI kidney transplantation. The pyramid highlights modifiable or partially modifiable contributors, including CIT, use of machine perfusion, and donor‐recipient matching, offering pragmatic opportunities to optimize organ utilization and post‐transplant outcomes.

**TABLE 4:** Ideal and suboptimal profiles for high‐KDPI kidney transplantation derived from adjusted‐risk predictors.

Evidence level	Domain	Ideal conditions for high‐KDPI kidney	Suboptimal conditions for high‐KDPI kidney
**Recipient‐level**	**Physiological reserve**	Preserved functional status (Karnofsky >70%)	Poor functional status (Karnofsky ≤ 70%)
		BMI 18.5–29.9 kg/m^2^	Diabetes and/or peripheral vascular disease
		Absence of advanced comorbidity (no diabetes, no peripheral vascular disease)	Severe obesity or underweight status
**Recipient‐level**	**Dialysis exposure**	Dialysis duration < 5 years	Dialysis duration > 5 years (higher risk of DGF, graft loss, and mortality)
		Greatest benefit in recipients with < 3 years of dialysis	
**Recipient‐level**	**Recipient age & resilience**	Age ≥ 35 years with preserved functional status	Age < 35 years (higher immunologic activity, adherence concerns)
		Ability to tolerate early post‐transplant morbidity	Frailty or limited physiological reserve
**Immunologic‐level**	**Viral‐immunologic risk**	Low‐ or intermediate‐risk CMV/EBV serostatus	CMV D+/R− or EBV D+/R− serostatus (increased risk of PGNF and graft loss)
		Avoidance of high‐risk D+/R− pairings when possible	
**Donor‐level**	**Donor renal function & injury phenotype**	Terminal donor creatinine ≤ 2.0 mg/dL or improving trajectory	Terminal donor creatinine > 2.0 mg/dL with worsening or non‐recovering trajectory
		Preserved or recovering urine output	Oliguria or anuria prior to procurement
		Hemodynamically stable donor (vasopressor‐supported but responsive)	Prolonged or refractory hemodynamic instability
		Renal dysfunction consistent with reversible ischemic or hemodynamic injury	Injury phenotype suggestive of severe or sustained acute kidney injury
**Donor‐level**	**Composite donor risk alignment**	Absence of multiple converging donor risk factors	Donation after circulatory death combined with high creatinine, prolonged ischemia, or poor perfusion metrics
		Donation after circulatory death without additional major injury signals	
**Graft‐level**	**Preservation & perfusion quality**	Machine perfusion used when available	No machine perfusion
		Favorable perfusion parameters (RI ≤ 0.4)	Elevated intrarenal resistive index (> 0.4)
**Logistical‐level**	**Ischemic exposure**	Cold ischemia time < 24 h	Cold ischemia time ≥ 24 h
		Expedited allocation and acceptance pathways	Expected delays in placement or prolonged transport

## 4 Discussion

One of the central challenges in transplant decision‐making is determining which organs can be safely utilized and for which candidates [22]. Although prior studies have demonstrated that transplantation with high‐ KDPI kidneys can confer a survival advantage compared with remaining on dialysis [12], these organs continue to be declined at disproportionately high rates [6]. This persistent underutilization reflects a fundamental clinical dilemma: high‐KDPI kidneys represent a potentially life‐saving resource for patients with ESRD, yet they are associated with increased risks of DGF, early graft loss, and post‐transplant morbidity [12]. At the time of organ offer, clinicians must weigh these competing considerations under conditions of time pressure, incomplete information, and increasing scrutiny of early post‐transplant outcomes. Despite the central role of high‐KDPI kidneys in contemporary allocation, there has been limited national evidence to clarify which donor, recipient, immunologic, and logistical factors most strongly drive outcomes within this highest‐risk donor pool. The present national analysis was undertaken to address this gap.

This study provides clinically informative insights that extend prior work on high‐KDPI kidney transplantation. Whereas earlier studies largely focused on demonstrating aggregate survival benefit or evaluating individual risk factors in isolation, this analysis specifically examined outcomes within the KDPI > 85% population and integrated multiple risk domains into an offer‐time, clinically interpretable framework. In doing so, it clarifies why KDPI alone loses discriminatory utility at the upper end of the spectrum and identifies which non‐KDPI factors meaningfully shape outcomes once the high‐risk threshold is crossed. Consistent with prior reports, transplantation with high‐KDPI kidneys was associated with acceptable outcomes when appropriately matched and managed, and ischemia‐reperfusion injury remained a central determinant of early graft dysfunction [3, 6, 11, 17]. However, outcomes did not differ meaningfully across KDPI strata of 86%–90%, 91%–95%, and > 95%, indicating that the KDPI percentile itself offers limited discriminatory value within this range [12, 22].

Within this context, donor and organ‐level physiology emerged as dominant determinants of early graft performance. Donation after circulatory death, elevated terminal serum creatinine, and abnormal intrarenal resistance during machine perfusion were strongly associated with DGF, PGNF, and inferior graft survival. These findings reinforce prior mechanistic and clinical studies demonstrating that dynamic indicators of renal injury and perfusion integrity better capture vulnerability to ischemia‐reperfusion injury than static donor descriptors alone [17, 18]. Importantly, within the high‐KDPI population, the cumulative burden of ischemic and physiologic stress—rather than donor age or KDPI percentile per se—appeared to drive early graft dysfunction.

The observed association between donor age younger than 35 years and inferior outcomes further underscores the importance of interpreting donor characteristics within their broader injury phenotype. This finding should not be interpreted as younger donor age being intrinsically harmful. Rather, donors younger than 35 years who meet criteria for KDPI > 85% represent a highly selected subgroup characterized by clustered adverse features, including severe acute kidney injury, profound hemodynamic instability, or high‐risk causes of death. In this setting, younger age functions as a marker of extreme donor injury rather than a protective attribute, challenging conventional assumptions and highlighting the limitations of interpreting individual donor variables in isolation [23, 24, 25, 26].

Logistical and technical factors, many of which are modifiable at the system level, were among the most consistent predictors of adverse outcomes. Prolonged CIT was strongly associated with DGF, graft loss, and mortality, reinforcing extensive prior evidence linking ischemic exposure to early graft injury [17, 27, 28]. The high frequency of CIT exceeding 24 h in this cohort suggests that a substantial component of ischemic risk is not biologically inevitable but reflects inefficiencies in allocation and acceptance workflows. These findings highlight opportunities for improvement through expedited placement strategies, including early identification of candidates willing to accept high‐KDPI kidneys and streamlined offer‐acceptance processes. Similarly, machine perfusion was associated with a lower risk of DGF yet was used in fewer than half of cases, despite near‐universal biopsy, identifying an immediately actionable target for mitigating ischemia‐reperfusion injury in marginal grafts [17, 29, 30].

Recipient‐level characteristics exerted an equally important influence on outcomes. Dialysis duration emerged as one of the strongest predictors across patient survival and graft endpoints, with a graded increase in risk beyond three years and particularly beyond five years of dialysis exposure. These findings confirm prior work linking prolonged dialysis to inferior post‐transplant outcomes while extending existing knowledge by demonstrating that dialysis vintage remains a dominant determinant even within the high‐KDPI setting [12, 31]. Clinically, this directly informs the long‐standing question of which candidates are most likely to benefit from high‐KDPI kidneys. Rather than default allocation to the sickest or most comorbid patients, the data support prioritizing recipients with preserved physiological reserve, shorter dialysis exposure, and manageable comorbidity burden who can tolerate early complications such as DGF, which affected more than one‐third of recipients in this cohort. This refines earlier paradigms that emphasized limited post‐transplant life expectancy by highlighting physiological resilience and dialysis exposure as more discriminating offer‐time signals than chronological age alone [12, 22].

Consistent with this framework, older recipients often represent appropriate candidates for high‐KDPI kidneys, particularly given the high mortality associated with prolonged dialysis in this population [12, 31, 32, 33]. Age greater than 65 years should therefore not be viewed as a contraindication but rather considered alongside functional status and dialysis vintage. Conversely, recipients with prolonged dialysis exposure, marked frailty, or severe comorbidity may be less able to withstand the morbidity associated with high‐risk grafts, potentially eroding any survival benefit. Viral‐immunologic configuration also contributed to outcome heterogeneity. Associations between CMV and Epstein‐Barr virus serologic mismatch and graft outcomes persisted in adjusted models and, although not necessarily causal, suggest that viral‐immunologic vulnerability may amplify susceptibility to early graft stress and warrants further investigation.

Taken together, these findings support a shift away from binary acceptance decisions based solely on KDPI thresholds toward a multidomain, offer‐time assessment that integrates donor injury phenotype, ischemic exposure, recipient physiological reserve, immunologic configuration, and logistical execution. The Integrated Risk Pyramid derived from this analysis and proposed as a guide at the moment of organ offer is not intended to function as a new allocation algorithm or a patient‐specific survival calculator. Rather, it provides an evidence‐informed framework that mirrors real‐world clinical reasoning while quantifying the relative contribution of key risk domains within the high‐KDPI setting. By explicitly distinguishing modifiable factors—such as CIT and machine perfusion—from recipient selection signals, this approach offers a pragmatic pathway to improving kidney utilization without compromising accountability for outcomes.

This analysis has limitations. Because only transplanted kidneys were examined, the findings may not fully reflect the characteristics or outcomes of high‐KDPI organs that were repeatedly declined or ultimately discarded. Registry data lack granular information on donor hemodynamic trajectories, standardized biopsy interpretation—particularly vascular pathology—frailty and sarcopenia, immunosuppression management, and intraoperative complexity. Warm ischemia time and anastomotic duration were not reliably captured, and the registry does not consistently distinguish among donation‐after‐circulatory‐death procurement strategies. In addition, because this cohort spans 2014–2021, donor characteristics and allocation practices have continued to evolve, and extrapolation to contemporary practice should be made with caution. These limitations highlight important directions for future research, including prospective studies incorporating detailed perfusion metrics, standardized biopsy interpretation, granular immunological profiling, and evaluation of system‐level interventions aimed at improving utilization without adverse effects on outcomes.

In conclusion, outcomes after transplantation of kidneys with KDPI greater than 85% are determined not by the KDPI percentile alone but by the interaction of donor injury phenotype, ischemic and logistical stress, recipient physiologic reserve, and immunologic configuration. When these domains are favorably aligned—particularly in recipients with preserved functional status, shorter dialysis exposure, and minimized ischemic injury—high‐KDPI kidneys can provide durable and clinically meaningful benefit. By clarifying which non‐KDPI factors matter most at the time of offer, this work addresses a central source of clinical uncertainty and provides a pragmatic foundation for more consistent, evidence‐informed acceptance decisions that may reduce avoidable discard while maintaining outcome accountability.

## Conflicts of Interest

The author declares no conflicts of interest.

## Supporting information




**Table S1**. Overview of Scientific Registry of Transplant Recipients (SRTR) variables extracted for the study and their operational definitions.
**Table S2**. Variables Included in the Multivariable Regression Models and Corresponding Reference Categories. Check marks (✓) indicate inclusion of the variable in each multivariable model evaluating primary graft non‐function (PGNF), delayed graft function (DGF), patient survival, graft survival, and death‐censored graft survival. Reference categories used to compute adjusted odds ratios and adjusted hazard ratios are listed in parentheses.
**Table S3**. Extent of Missing Data for Donor, Recipient, Immunologic, and Perioperative Variables. Number and percentage of missing observations for variables extracted from the Scientific Registry of Transplant Recipients (SRTR). Warm ischemia time was incompletely reported during the study period and was excluded from primary multivariable analyses.
**Table S4**. Unadjusted odds ratios for delayed graft function (DGF) in recipients of High‐KDPI kidneys.
**Table S5**. Unadjusted odds ratios for primary graft non‐function in recipients of High‐KDPI kidneys.
**Table S6**. Unadjusted hazard ratio for the risk of death after renal transplantation with high‐KDPI kidneys.
**Table S7**. Unadjusted 1‐, 3‐, and 5‐year graft survival rates of high‐KDPI kidneys across different recipient and donor subgroups.
**Table S7** Unadjusted hazard ratio for the risk of graft loss after renal transplantation with high‐KDPI kidneys.
**Table S8** Unadjusted hazard ratio for the risk of death‐censored graft loss after renal transplantation with high‐KDPI kidneys.
**Table S9. Sensitivity Analysis: Multivariable Logistic Regression Model for Delayed Graft Function Excluding Preemptively Transplanted Recipients**. This table presents results from a sensitivity analysis using a multivariable logistic regression model evaluating independent predictors of delayed graft function (DGF), defined as the need for dialysis within 7 days after transplantation, among recipients of kidneys with Kidney Donor Profile Index (KDPI) >85%. Analyses were restricted to recipients who underwent transplantation after initiation of dialysis, excluding those who were transplanted preemptively, to reduce potential outcome misclassification. Results are reported as regression coefficients, standard errors, adjusted odds ratios (aORs), 95% confidence intervals (CIs), and corresponding P values. Reference categories are indicated for categorical variables. All variables were selected a priori and entered simultaneously into the fully adjusted model.
**Table S10. Sensitivity Analysis: Multivariable Logistic Regression Model for Primary Graft Nonfunction Excluding Preemptively Transplanted Recipients**. This table presents results from a sensitivity analysis using a multivariable logistic regression model assessing independent predictors of primary graft nonfunction (PGNF), defined as irreversible allograft failure resulting in permanent dialysis dependence or graft nephrectomy within 90 days after transplantation, among recipients of kidneys with KDPI > 85%. Analyses were limited to recipients who underwent transplantation after dialysis initiation, excluding preemptively transplanted patients, to ensure robust classification of early graft failure. Results are shown as regression coefficients, standard errors, adjusted odds ratios (aORs), 95% confidence intervals (CIs), and P values. Reference categories are specified for categorical variables, and all covariates were selected a priori based on clinical relevance.
**Table S11. Sensitivity Analysis: Fine–Gray Competing‐Risk Regression of Factors Associated With Graft Failure Among Recipients of High‐KDPI Kidneys**. This table presents results from a sensitivity analysis using Fine–Gray competing‐risk regression to evaluate factors associated with kidney graft failure among recipients of kidneys with Kidney Donor Profile Index (KDPI) > 85%. The model estimates sub distribution hazard ratios (sHRs) for graft failure while treating death with a functioning graft as a competing event rather than a censoring event. Results are reported as regression coefficients, standard errors, sub distribution hazard ratios (sHRs), 95% confidence intervals (CIs), Z statistics, and corresponding P values. An sHR greater than 1 indicates a higher cumulative incidence of graft failure, whereas an sHR less than 1 indicates a lower cumulative incidence, after accounting for competing mortality. This analysis was performed to assess the robustness of findings from cause‐specific Cox models.

## Data Availability

The data that support the findings of this study are available on request from the corresponding author. The data are not publicly available due to privacy or ethical restrictions.
