# Supplementary material for: “Rethinking High‐KDPI Kidneys: A Multidomain Approach to Predicting Success”
Source: Clin Transplant. 2026 Feb 4;40(2):e70472. doi: 10.1111/ctr.70472 (PMC12872201; doi:10.1111/ctr.70472)
Supplement: Supplementary file 1 — Table S1. Overview of Scientific Registry of Transplant Recipients (SRTR) variables extracted for the study and their operational definitions. Table S2. Variables Included in the Multivariable Regression Models and Corresponding Reference Categories. Check marks (✓) indicate inclusion of the variable in each multivariable model evaluating primary graft non‐function (PGNF), delayed graft function (DGF), patient survival, graft survival, and death‐censored graft survival. Reference categories used to compute adjusted odds ratios and adjusted hazard ratios are listed in parentheses. Table S3. Extent of Missing Data for Donor, Recipient, Immunologic, and Perioperative Variables. Number and percentage of missing observations for variables extracted from the Scientific Registry of Transplant Recipients (SRTR). Warm ischemia time was incompletely reported during the study period and was excluded from primary multivariable analyses. Table S4. Unadjusted odds ratios for delayed graft function (DGF) in recipients of High‐KDPI kidneys. Table S5. Unadjusted odds ratios for primary graft non‐function in recipients of High‐KDPI kidneys. Table S6. Unadjusted hazard ratio for the risk of death after renal transplantation with high‐KDPI kidneys. Table S7. Unadjusted 1‐, 3‐, and 5‐year graft survival rates of high‐KDPI kidneys across different recipient and donor subgroups. Table S7 Unadjusted hazard ratio for the risk of graft loss after renal transplantation with high‐KDPI kidneys. Table S8 Unadjusted hazard ratio for the risk of death‐censored graft loss after renal transplantation with high‐KDPI kidneys. Table S9. Sensitivity Analysis: Multivariable Logistic Regression Model for Delayed Graft Function Excluding Preemptively Transplanted Recipients. This table presents results from a sensitivity analysis using a multivariable logistic regression model evaluating independent predictors of delayed graft function (DGF), defined as the need for dialysis within 7 days after transplanta [file CTR-40-e70472-s001.docx]

**Supplementary Material**

**Table S1.** Overview of Scientific Registry of Transplant Recipients (SRTR) variables extracted for the study and their operational definitions.

| **Variable** | **Stratification Categories** |
| --- | --- |
| Age | <35, 35–49, 50–64, ≥65 years |
| Sex | Male, Female |
| Ethnicity | White – Black – Hispanic- Asian - Other |
| BMI | Underweight (<18.5), Normal (18.5–24.9), Overweight (25–29.9), Obese (≥30) |
| History of diabetes | Yes, No |
| Peripheral vascular disease | Yes, No |
| Etiology of renal disease | Diabetes - hypertension – glomerulitis - other |
| c-PRA | 0–84%, 85–90%, 91–94%, ≥95% |
| Functional status | Poor (<50), Fair (50-70), Good (>70) |
| KDPI group | 86–90%, 91–95%, >95% |
| Dialysis duration | <3 years, 3–5 years, >5 years |
| EBV risk | Low (D–/R–), Intermediate (D+/R+ or D–/R+), High (D+/R–) |
| CMV risk | Low (D–/R–), Intermediate (D+/R+ or D–/R+), High (D+/R–) |
| HLA mismatches | 0–3, 4, 5, 6 |
| Donor age | <35, 35–49, 50–64, ≥65 years |
| Type of donation | Brain death, Circulatory death |
| Proteinuria | Yes, No |
| Peak creatinine | <1.0, 1.0–1.5, 1.51–2.0, >2.0 mg/dL |
| Terminal sodium | <135, 135–145, >145 mEq/L |
| CDC high-risk status | Yes, No |
| Use of vasopressors | Yes, No |
| Biopsy performed | Yes, No |
| Glomerulosclerosis % | 0–5%, 6–10%, 11–15%, 16–20%, >20% |
| Resistive Index of perfusion pump | 0–0.4, >0.4 |
| Cold Ischemia Time | 0–11.9, 12–17.9, 18–23.9, ≥24 hours |
| Delayed graft function | Yes, No |

**Legend.** Continuous variables were categorized using established clinical thresholds or distribution-based cut points relevant to kidney transplantation. These categorizations were applied uniformly across all bivariable and multivariable models. **Abbreviations:** BMI, body mass index; cPRA, calculated panel reactive antibody; KDPI, Kidney Donor Profile Index; EBV, Epstein–Barr virus; CMV, cytomegalovirus; HLA, human leukocyte antigen; CDC, Centers for Disease Control and Prevention.

**Table S2.** Variables Included in the Multivariable Regression Models and Corresponding Reference Categories. Check marks (✓) indicate inclusion of the variable in each multivariable model evaluating primary graft non-function (PGNF), delayed graft function (DGF), patient survival, graft survival, and death-censored graft survival. Reference categories used to compute adjusted odds ratios and adjusted hazard ratios are listed in parentheses.

| **Variable (Reference Category)** | **PGNF** | **DGF** | **Patient Survival** | **Graft Survival** | **Death-Censored Graft Survival** |
| --- | --- | --- | --- | --- | --- |
| Age (≥65 years) | ✓ | ✓ | ✓ | ✓ | ✓ |
| Sex (Female) | ✓ | ✓ | ✓ | ✓ | ✓ |
| Ethnicity (White) | ✓ | ✓ | ✓ | ✓ | ✓ |
| BMI (Normal) | ✓ | ✓ | ✓ | ✓ | ✓ |
| History of diabetes (No diabetes) | ✓ | ✓ | ✓ | ✓ | ✓ |
| Peripheral vascular disease (No PVD) | ✓ | ✓ | ✓ | ✓ | ✓ |
| Etiology of renal disease (Hypertension) | ✓ | ✓ | ✓ | ✓ | ✓ |
| cPRA (0–84%) | ✓ | ✓ | ✓ | ✓ | ✓ |
| Functional status (Good) | ✓ | ✓ | ✓ | ✓ | ✓ |
| KDPI group (86–90%) | ✓ | ✓ | ✓ | ✓ | ✓ |
| Dialysis duration (0–3 years) | ✓ | ✓ | ✓ | ✓ | ✓ |
| EBV risk (Intermediate) | ✓ | ✓ | ✓ | ✓ | ✓ |
| CMV risk (Intermediate) | ✓ | ✓ | ✓ | ✓ | ✓ |
| HLA mismatches (0–3) | ✓ | ✓ | ✓ | ✓ | ✓ |
| Donor age (≥65 years) | ✓ | ✓ | ✓ | ✓ | ✓ |
| Type of donation (Brain-dead) | ✓ | ✓ | ✓ | ✓ | ✓ |
| Proteinuria (No proteinuria) | ✓ | ✓ | ✓ | ✓ | ✓ |
| Peak creatinine (1.0–1.5 mg/dL) | ✓ | ✓ | ✓ | ✓ | ✓ |
| Terminal sodium (135–145 mEq/L) | ✓ | ✓ | ✓ | ✓ | ✓ |
| CDC high-risk (No high risk) | ✓ | ✓ | ✓ | ✓ | ✓ |
| Use of vasopressors (None) | ✓ | ✓ | ✓ | ✓ | ✓ |
| Biopsy performed (Yes) | ✓ | ✓ | ✓ | ✓ | ✓ |
| Glomerulosclerosis % (0–5%) | ✓ | ✓ | ✓ | ✓ | ✓ |
| Use of perfusion pump (Pump used) | ✓ | ✓ | ✓ | ✓ | ✓ |
| Resistive index (<0.40) | ✓ | ✓ | ✓ | ✓ | ✓ |
| Cold ischemia time (0–11.9 hours) | ✓ | ✓ | ✓ | ✓ | ✓ |
| Delayed graft function (No DGF) | - | - | ✓ | ✓ | ✓ |

**Supplementary Table S3.** Extent of Missing Data for Donor, Recipient, Immunologic, and Perioperative Variables. Number and percentage of missing observations for variables extracted from the Scientific Registry of Transplant Recipients (SRTR). Warm ischemia time was incompletely reported during the study period and was excluded from primary multivariable analyses.

| **Variable** | | | **Missed Value, n. (%)** | |
| --- | --- | --- | --- | --- |
| **Recipient factors** | Age | 0 | |  |
|  | Sex | 0 | |  |
|  | Ethnicity | 0 | |  |
|  | BMI | 95 (1.9) | |  |
|  | History of diabetes | 1 (0.02) | |  |
|  | History of peripheral vascular disease | 0 | |  |
|  | Cause of renal disease | 333 (6.7) | |  |
|  | cPRA | 1 (0.02) | |  |
|  | Functional status | 136 (2.8) | |  |
|  | KDPI | 0 | |  |
|  | Dialysis duration | 45 (0.9) | |  |
|  | EBV serostatus | 314 (6.4) | |  |
|  | CMV serostatus | 73 (1.5) | |  |
|  | HLA mismatches | 0 | |  |
| **Donor factors** | Age | 0 | |  |
|  | Sex | 0 | |  |
|  | Ethnicity | 0 | |  |
|  | BMI | 0 | |  |
|  | Type of donation | 0 | |  |
|  | Proteinuria | 0 | |  |
|  | Peak serum creatinine | 0 | |  |
|  | Terminal sodium | 0 | |  |
|  | CDC high-risk status | 0 | |  |
|  | Use of vasopressors | 0 | |  |
| **Graft/preservation factors** | Biopsy performed (yes/no) | 0 | |  |
|  | Glomerulosclerosis % | 274 (5.6) | |  |
|  | Resistive Index for grafts on perfusion pump | 0 | |  |
| **Logistical factors** | Duration of cold ischemia | 77 (1.5) | |  |
|  | Duration of warm ischemia | 2726 (55.5) | |  |
| **Short-term Outcomes** | Delayed graft function | 0 | |  |

**Abbreviations:** BMI, body mass index; cPRA, calculated panel reactive antibody; KDPI, Kidney Donor Profile Index; EBV, Epstein–Barr virus; CMV, cytomegalovirus; HLA, human leukocyte antigen; CDC, Centers for Disease Control and Prevention.

**Table S4.** Unadjusted odds ratios for delayed graft function (DGF) in recipients of High-KDPI kidneys.

| **Characteristics** | **Unadjusted Odds Ratio** | **P value** |
| --- | --- | --- |
| **Age** |  |  |
| >65 yrs | Reference [1] |  |
| <35 yrs | 0.71(0.23-2.22) | 0.558 |
| 35-49 yrs | 0.95 (0.72-1.26) | 0.734 |
| 50-64 yrs | 1.23 (1.09-1.39) | <0.001 |
| **Sex** |  |  |
| Female | Reference [1] |  |
| Male | 1.47 (1.30-1.67) | <0.001 |
| **Race-ethnicity** |  |  |
| White-Caucasian | Reference [1] |  |
| Black-African American | 1.89 (1.63-2.18) | <0.001 |
| Hispanic-Latino | 1.82 (1.54-2.16) | <0.001 |
| Asian | 1.77 (1.41-2.21) | <0.001 |
| Other | 1.41 (0.84-2.37) | 0.196 |
| **Body Mass Index (BMI)** |  |  |
| 18.5-24.9 | Reference [1] |  |
| <18.5 | 0.64 (0.42-0.98) | 0.039 |
| 25-29.9 | 1.26 (1.08-1.48) | 0.003 |
| ≥ 30 | 1.68 (1.44-1.96) | <0.001 |
| **History of diabetes** |  |  |
| NO | Reference [1] |  |
| YES | 1.40 (1.24-1.57) | <0.001 |
| **History of peripheral vascular disease** |  |  |
| NO | Reference [1] |  |
| YES | 1.32 (1.12-1.55) | 0.009 |
| **Etiology of Renal Failure** |  |  |
| Diabetic nephropathy | Reference [1] |  |
| Hypertension | 0.82 (0.71-0.95) | 0.007 |
| Glomerulonephritis | 0.64 (0.52-0.79) | <0.001 |
| Other | 0.58 (0.47-0.72) | <0.001 |
| **c-PRA (%)** |  |  |
| 0-84 | Reference [1] |  |
| 85-90 | 1.73 (0.67-4.49) | 0.260 |
| 90-94 | 0.51 (0.19-1.38) | 0.184 |
| ≥ 95 | 0.72 (0.45-1.15) | 0.169 |
| **Karnofsky Functional Index, n. (%)** |  |  |
| <50 (poor) | Reference [1] |  |
| 50-70 (fair) | 0.97 (0.65-1.44) | 0.875 |
| >70 (good) | 0.80(0.54-1.19) | 0.272 |
| **KDPI Group** |  |  |
| 86-90 | Reference [1] |  |
| 91-95 | 0.89 (0.78-1.02) | 0.097 |
| >95 | 0.91 (0.77-1.07) | 0.229 |
| **Dialysis duration** |  |  |
| 0-3 years, n. (%) | Reference [1] |  |
| 3.1-5 years, n. (%) | 1.74 (1.49-2.03) | <0.001 |
| > 5 years, n. (%) | 2.31 (2.01-2.66) | <0.001 |
| **EBV status** |  |  |
| Intermediate risk | Reference [1] |  |
| Low risk | 1.53 (0.41-5.72) | 0.524 |
| High risk | 0.84 (0.64-1.11) | 0.225 |
| **CMV status** |  |  |
| Intermediate risk | Reference [1] |  |
| Low risk | 0.84 (0.67-1.04) | 0.109 |
| High risk | 0.81 (0.70-0.94) | 0.006 |
| **HLA mismatch** |  |  |
| 0-3 | Reference [1] |  |
| 4 | 1.12 (0.91-1.37) | 0.278 |
| 5 | 1.08 (0.89-1.30) | 0.447 |
| 6 | 1.14 (0.93-1.39) | 0.217 |
| **Age** |  |  |
| >65 yrs | Reference [1] |  |
| <35 yrs | 1.28 (0.55-2.99) | 0.562 |
| 35-49 yrs | 1.03 (0.75-1.41) | 0.857 |
| 50-64 yrs | 1.03 (0.90-1.17) | 0.677 |
| **Cardiac Death** |  |  |
| NO | Reference [1] |  |
| YES | 2.45 (2.01-3.00) | <0.001 |
| **Proteinuria** |  |  |
| NO | Reference [1] |  |
| YES | 1.17 (1.04-1.32) | 0.009 |
| **Peak serum creatinine** |  |  |
| <1.0 | Reference [1] |  |
| 1.0-1.5 | 1.13 (0.95-1.33) | 0.161 |
| 1.51-2.0 | 1.49 (1.24-1.80) | <0.001 |
| > 2.0 | 1.62 (1.33-1.98) | <0.001 |
| **Terminal sodium** |  |  |
| <135 | Reference [1] |  |
| 135-145 | 1.16 (0.77-1.75) | 0.483 |
| >145 | 1.20 (0.80-1.80) | 0.375 |
| **CDC high risk** |  |  |
| NO | Reference [1] |  |
| YES | 0.88 (0.73-1.06) | 0.185 |
| **Need of vasopressor for hemodynamic stability** |  |  |
| NO | Reference [1] |  |
| YES | 0.76 (0.67-0.85) | <0.001 |
| **Biopsy performed** |  |  |
| NO | Reference [1] |  |
| YES | 0.87 (0.67-1.13) | 0.288 |
| 0-5% | Reference [1] |  |
| 6-10% | 1.08 (0.93-1.26) | 0.296 |
| 11-15% | 1.11 (0.90-1.38) | 0.313 |
| 16-20% | 0.83 (0.62-1.10) | 0.196 |
| >20% | 0.93 (0.67-1.29) | 0.658 |
| Unclear | 2.68 (0.60-11.99) | 0.198 |
| **Kidneys put on perfusion pump** |  |  |
| NO | Reference [1] |  |
| YES | 0.62 (0.55-0.70) | <0.001 |
| ≤0.4 | Reference [1] |  |
| >0.4 | 1.04 (0.69-1.56) | 0.869 |
| Missing | 1.59 (1.41-1.80) | <0.001 |
| **Cold ischemia time** |  |  |
| < 12 hours | Reference [1] |  |
| 12-17.9 hours | 1.11 (0.91-1.35) | 0.294 |
| 18-23.9 hours | 1.32 (1.09-1.60) | 0.004 |
| ≥ 24 hours | 1.29 (1.07-1.57) | 0.008 |

**Abbreviations:** BMI, body mass index; cPRA, calculated panel reactive antibody; KDPI, Kidney Donor Profile Index; EBV, Epstein–Barr virus; CMV, cytomegalovirus; HLA, human leukocyte antigen; CDC, Centers for Disease Control and Prevention.

**Table S5.** Unadjusted odds ratios for primary graft non-function in recipients of High-KDPI kidneys.

| **Characteristics** | **Unadjusted Odds Ratio** | **P value** |
| --- | --- | --- |
| **Age** |  |  |
| >65 yrs | Reference [1] |  |
| <35 yrs | 5.42 (1.21-24.35) | 0.0274 |
| 35-49 yrs | 1.24 (0.59-2.61) | 0.573 |
| 50-64 yrs | 1.21 (0.85-1.71) | 0.2917 |
| **Sex** |  |  |
| Female | Reference [1] |  |
| Male | 1.14 (0.80-1.63) | 0.4671 |
| **Race-ethnicity** |  |  |
| White-Caucasian | Reference [1] |  |
| Black-African American | 1.17 (0.80-1.71) | 0.4172 |
| Hispanic-Latino | 0.58 (0.34-1.01) | 0.0561 |
| Asian | 0.78 (0.39-1.55) | 0.4824 |
| Other | 0.48 (0.07-3.55) | 0.4748 |
| **Body Mass Index (BMI)** |  |  |
| 18.5-24.9 | Reference [1] |  |
| <18.5 | 0.30 (0.04-2.22) | 0.2389 |
| 25-29.9 | 1.23 (0.76-1.98) | 0.3926 |
| ≥ 30 | 1.68 (1.06-2.65) | 0.0259 |
| **History of diabetes** |  |  |
| NO | Reference [1] |  |
| YES | 0.94 (0.67-1.31) | 0.7029 |
| **History of peripheral vascular disease** |  |  |
| NO | Reference [1] |  |
| YES | 1.25 (0.81-1.95) | 0.3132 |
| **Etiology of Renal Failure** |  |  |
| Diabetic nephropathy | Reference [1] |  |
| Hypertension | 0.83 (0.53-1.29) | 0.4114 |
| Glomerulonephritis | 1.25 (0.74-2.11) | 0.406 |
| Other | 1.25 (0.74-2.11) | 0.4021 |
| **c-PRA (%)** |  |  |
| 0-84 | Reference [1] |  |
| 85-90 | 2.10 (0.28-15.97) | 0.4725 |
| 90-94 | - | 0.9804 |
| ≥ 95 | 0.38 (0.05-2.76) | 0.3407 |
| **Karnofsky Functional Index, n. (%)** |  |  |
| <50 (poor) | Reference [1] |  |
| 50-70 (fair) | 0.62(0.24-1.56) | 0.3073 |
| >70 (good) | 0.60(0.24-1.53) | 0.284 |
| **KDPI Group** |  |  |
| 86-90 | Reference [1] |  |
| 91-95 | 0.93 (0.64-1.35) | 0.6889 |
| >95 | 0.79 (0.49-1.28) | 0.3327 |
| **Dialysis duration** |  |  |
| 0-3 years, n. (%) | Reference [1] |  |
| 3.1-5 years, n. (%) | 1.15 (0.73-1.79) | 0.5551 |
| > 5 years, n. (%) | 1.47 (0.99-2.17) | 0.0541 |
| **EBV status** |  |  |
| Intermediate risk | Reference [1] |  |
| Low risk | - | 0.9816 |
| High risk | 1.06 (0.52-2.20) | 0.8669 |
| **CMV status** |  |  |
| Intermediate risk | Reference [1] |  |
| Low risk | 1.14 (0.62-2.10) | 0.6751 |
| High risk | 1.58 (1.08-2.33) | 0.0193 |
| **HLA mismatch** |  |  |
| 0-3 | Reference [1] |  |
| 4 | 1.66 (0.88-3.14) | 0.1167 |
| 5 | 1.50 (0.82-2.77) | 0.1921 |
| 6 | 1.38 (0.72-2.63) | 0.3353 |
| **Age** |  |  |
| >65 yrs | Reference [1] |  |
| <35 yrs | 9.89 (3.51-27.90) | <.0001 |
| 35-49 yrs | 0.56 (0.17-1.82) | 0.3345 |
| 50-64 yrs | 1.05 (0.72-1.51) | 0.8182 |
| **Cardiac Death** |  |  |
| NO | Reference [1] |  |
| YES | 1.93 (1.20-3.11) | 0.0065 |
| **Proteinuria** |  |  |
| NO | Reference [1] |  |
| YES | 0.88 (0.63-1.24) | 0.4726 |
| **Peak serum creatinine** |  |  |
| <1.0 | Reference [1] |  |
| 1.0-1.5 | 0.89 (0.55-1.45) | 0.651 |
| 1.51-2.0 | 1.16 (0.68-1.98) | 0.5957 |
| > 2.0 | 1.74 (1.03-2.93) | 0.0383 |
| **Terminal sodium** |  |  |
| <135 | Reference [1] |  |
| 135-145 | 0.54 (0.24-1.22) | 0.1374 |
| >145 | 0.40 (0.18-0.89) | 0.0254 |
| **CDC high risk** |  |  |
| NO | Reference [1] |  |
| YES | 0.83 (0.48-1.45) | 0.5151 |
| **Need of vasopressor for hemodynamic stability** |  |  |
| NO | Reference [1] |  |
| YES | 0.61 (0.43-0.86) | 0.0052 |
| **Biopsy performed** |  |  |
| NO | Reference [1] |  |
| YES | 0.52 (0.29-0.93) | 0.0263 |
| **Percentage of glomerulosclerosis identified in the surgical pathology specimen** |  |  |
| 0-5% | Reference [1] |  |
| 6-10% | 1.13 (0.73-1.73) | 0.5824 |
| 11-15% | 1.15 (0.63-2.09) | 0.6511 |
| 16-20% | 1.66 (0.85-3.27) | 0.1409 |
| >20% | 0.44 (0.11-1.80) | 0.2534 |
| Unclear | - | 0.9845 |
| **Kidneys put on perfusion pump** |  |  |
| NO | Reference [1] |  |
| YES | 0.82 (0.58-1.16) | 0.2701 |
| **Resistive index of kidneys while on perfusion pump** |  |  |
| ≤0.4 | Reference [1] |  |
| >0.4 | 2.52 (1.12-5.66) | 0.0256 |
| Missing | 1.24 (0.87-1.76) | 0.2337 |
| **Cold ischemia time** |  |  |
| < 12 hours | Reference [1] |  |
| 12-17.9 hours | 0.73 (0.43-1.22) | 0.2252 |
| 18-23.9 hours | 0.81 (0.50-1.34) | 0.4154 |
| ≥ 24 hours | 0.77 (0.47-1.28) | 0.3209 |

**Abbreviations:** BMI, body mass index; cPRA, calculated panel reactive antibody; KDPI, Kidney Donor Profile Index; EBV, Epstein–Barr virus; CMV, cytomegalovirus; HLA, human leukocyte antigen; CDC, Centers for Disease Control and Prevention.

**Table S6.** Unadjusted hazard ratio for the risk of death after renal transplantation with high-KDPI kidneys.

| **Characteristics** | **Unadjusted Hazard Ratio** | **P value** |
| --- | --- | --- |
| **Recipient Age** |  |  |
| >65 yrs | Reference [1] |  |
| <35 yrs | 1.29(0.58-2.88) | 0.539 |
| 35-49 yrs | 0.73(0.55-0.97) | 0.032 |
| 50-64 yrs | 0.95(0.85-1.07) | 0.416 |
| **Recipient Sex** |  |  |
| Female | Reference [1] |  |
| Male | 1.16(1.03-1.31) | 0.016 |
| **Recipient Race-ethnicity** |  |  |
| White-Caucasian | Reference [1] |  |
| Black-African American | 1.04(0.91-1.19) | 0.595 |
| Hispanic-Latino | 0.94(0.80-1.10) | 0.435 |
| Asian | 0.73(0.57-0.93) | 0.011 |
| Other | 0.74(0.41-1.34) | 0.321 |
| **Recipient Body Mass Index (BMI)** |  |  |
| 18.5-24.9 | Reference [1] |  |
| <18.5 | 1.05(0.69-1.60) | 0.806 |
| 25-29.9 | 1.02(0.88-1.19) | 0.773 |
| ≥ 30 | 1.12(0.96-1.30) | 0.142 |
| **Recipient History of diabetes** |  |  |
| NO | Reference [1] |  |
| YES | 1.30(1.15-1.46) | <.0001 |
| **Recipient History of peripheral vascular disease** | | |
| NO | Reference [1] |  |
| YES | 1.37(1.18-1.59) | <.0001 |
| **Etiology of Renal Failure** |  |  |
| Diabetic nephropathy | Reference [1] |  |
| Hypertension | 0.77(0.67-0.89) | 0.000 |
| Glomerulonephritis | 0.77(0.63-0.94) | 0.010 |
| Other | 0.75(0.61-0.92) | 0.006 |
| **c-PRA (%)** |  |  |
| 0-84 | Reference [1] |  |
| 85-90 | 1.74(0.72-4.20) | 0.215 |
| 90-94 | 0.19(0.03-1.37) | 0.101 |
| ≥ 95 | 0.91(0.60-1.39) | 0.661 |
| **Karnofsky Functional Index, n. (%)** |  |  |
| <50 (poor) | Reference [1] |  |
| 50-70 (fair) | 0.66(0.47-0.91) | 0.011 |
| >70 (good) | 0.49(0.36-0.68) | <.0001 |
| **KDPI Group** |  |  |
| 86-90 | Reference [1] |  |
| 91-95 | 1.03(0.90-1.17) | 0.711 |
| >95 | 1.12(0.95-1.31) | 0.180 |
| **Dialysis duration, years** |  |  |
| 0-3 | Reference [1] |  |
| 3.1-5 | 1.35(1.15-1.57) | 0.000 |
| > 5 | 1.44(1.25-1.65) | <.0001 |
| **EBV serous status** |  |  |
| Intermediate risk | Reference [1] |  |
| Low risk | 0.35(0.05-2.49) | 0.295 |
| High risk | 1.29(1.03-1.62) | 0.025 |
| **CMV serous status** |  |  |
| Intermediate risk | Reference [1] |  |
| Low risk | 0.94(0.75-1.17) | 0.547 |
| High risk | 1.21(1.05-1.39) | 0.009 |
| **HLA mismatch** |  |  |
| 0-3 | Reference [1] |  |
| 4 | 1.02(0.83-1.25) | 0.874 |
| 5 | 1.16(0.96-1.40) | 0.127 |
| 6 | 1.23(1.01-1.50) | 0.042 |
| **Donor Age** |  |  |
| >65 yrs | Reference [1] |  |
| <35 yrs | 1.71(0.91-3.20) | 0.096 |
| 35-49 yrs | 0.85(0.60-1.20) | 0.358 |
| 50-64 yrs | 1.11(0.98-1.26) | 0.105 |
| **Donation after cardiocirculatory arrest** |  |  |
| NO | Reference [1] |  |
| YES | 1.26(1.01-1.58) | 0.039 |
| **Donor Proteinuria** |  |  |
| NO | Reference [1] |  |
| YES | 0.98(0.87-1.10) | 0.702 |
| **Donor Peak serum creatinine, (mg/dL)** |  |  |
| <1.0 | Reference [1] |  |
| 1.0-1.5 | 1.01(0.87-1.18) | 0.872 |
| 1.51-2.0 | 0.95(0.78-1.14) | 0.550 |
| > 2.0 | 1.19(0.98-1.45) | 0.072 |
| **Donor Terminal serum sodium (mEq/L)** |  |  |
| <135 | Reference [1] |  |
| 135-145 | 0.80(0.56-1.15) | 0.224 |
| >145 | 0.77(0.54-1.09) | 0.139 |
| **CDC high risk status** |  |  |
| NO | Reference [1] |  |
| YES | 0.75(0.61-0.93) | 0.010 |
| **Need of vasopressor for donor hemodynamic stability** | |  |
| NO | Reference [1] |  |
| YES | 0.92(0.82-1.03) | 0.162 |
| **Biopsy performed** |  |  |
| NO | Reference [1] |  |
| YES | 1.05(0.79-1.39) | 0.753 |
| **Percentage of glomerulosclerosis identified in the surgical pathology specimen** | |  |
| 0-5% | Reference [1] |  |
| 6-10% | 1.08(0.93-1.24) | 0.316 |
| 11-15% | 1.06(0.86-1.31) | 0.563 |
| 16-20% | 1.14(0.88-1.49) | 0.321 |
| >20% | 0.96(0.70-1.32) | 0.807 |
| Immediate | 2.38(0.99-5.74) | 0.054 |
| **Kidneys put on perfusion pump** |  |  |
| NO | Reference [1] |  |
| YES | 1.01(0.90-1.14) | 0.811 |
| **Resistive index of kidneys while on perfusion pump** | | |
| ≤0.4 | Reference [1] |  |
| >0.4 | 1.45(1.02-2.05) | 0.037 |
| Missing | 1.01(0.90-1.14) | 0.864 |
| **Cold ischemia time, hours** |  |  |
| < 12 | Reference [1] |  |
| 12-17.9 | 1.12(0.92-1.37) | 0.243 |
| 18-23.9 | 1.21(1.00-1.46) | 0.054 |
| ≥ 24 | 1.20(0.99-1.45) | 0.065 |
| **Delayed Graft Function** |  |  |
| NO | Reference [1] |  |
| YES | 1.75(1.55-1.96) | <.0001 |

**Abbreviations:** BMI, body mass index; cPRA, calculated panel reactive antibody; KDPI, Kidney Donor Profile Index; EBV, Epstein–Barr virus; CMV, cytomegalovirus; HLA, human leukocyte antigen; CDC, Centers for Disease Control and Prevention.

**Table S7.** Unadjusted 1-, 3-, and 5-year graft survival rates of high-KDPI kidneys across different recipient and donor subgroups.

| **Characteristics** | **1-year** | **3-year** | **5-year** |
| --- | --- | --- | --- |
| **KDPI** | 0.89 (0.88-0.90) | 0.75 (0.72-0.78) | 0.61 (0.57-0.66) |
| 86-90 | 0.89 (0.88-0.90) | 0.75 (0.73-0.78) | 0.64 (0.61-0.66) |
| 91-95 | 0.89 (0.88-0.91) | 0.76 (0.74-0.79) | 0.62 (0.58-0.66) |
| >95 | 0.88 (0.86-0.90) | 0.74 (0.70-0.77) | 0.59 (0.54-0.65) |
| **Cold ischemic time (hours)** |  |  |  |
| 0-11.9 | 0.90 (0.88-0.92) | 0.77 (0.73-0.81) | 0.65 (0.59-0.70) |
| 12-17.9 | 0.89 (0.87-0.91) | 0.76 (0.73-0.79) | 0.64 (0.59-0.68) |
| 18-23.9 | 0.88 (0.86-0.90) | 0.75 (0.72-0.77) | 0.61 (0.57-0.65) |
| >=24 | 0.89 (0.87-0.91) | 0.74 (0.72-0.77) | 0.61 (0.57-0.65) |
| **Use of pulsatile perfusion pump** |  |  |  |
| No | 0.89 (0.88-0.90) | 0.76 (0.74-0.78) | 0.61 (0.59-0.64) |
| Yes | 0.88 (0.87-0.90) | 0.75 (0.72-0.77) | 0.64 (0.60-0.67) |
| **Arterial Resistive Index** |  |  |  |
| <0.4 | 0.89 (0.87-0.90) | 0.75 (0.73-0.78) | 0.64 (0.61-0.68) |
| >0.4 | 0.84 (0.77-0.91) | 0.67 (0.56-0.78) | 0.46 (0.29-0.63) |
| Missing | 0.89 (0.88-0.91) | 0.76 (0.74-0.78) | 0.61 (0.59-0.64) |
| **HLA mismatch** |  |  |  |
| 0-3 | 0.89 (0.87-0.92) | 0.79 (0.75-0.82) | 0.64 (0.58-0.70) |
| 4 | 0.90 (0.88-0.92) | 0.77 (0.74-0.80) | 0.66 (0.62-0.70) |
| 5 | 0.89 (0.88-0.91) | 0.74 (0.72-0.77) | 0.61 (0.58-0.64) |
| 6 | 0.88 (0.86-0.90) | 0.74 (0.71-0.77) | 0.59 (0.55-0.63) |
| **Recipient BMI (kg/m^2^)** |  |  |  |
| <=18.5 | 0.93 (0.88-0.98) | 0.75 (0.65-0.86) | 0.60 (0.45-0.76) |
| 18.5-24.9 | 0.90 (0.89-0.92) | 0.76 (0.73-0.79) | 0.63 (0.59-0.67) |
| 25-29.9 | 0.89 (0.87-0.90) | 0.75 (0.73-0.78) | 0.64 (0.61-0.68) |
| >30 | 0.88 (0.86-0.89) | 0.75 (0.72-0.77) | 0.60 (0.56-0.64) |
| **EBV** |  |  |  |
| low risk | 1.00 (1.00-1.00) | 0.88 (0.65-1.10) | 0.88 (0.65-1.10) |
| intermediate risk | 0.89 (0.88-0.90) | 0.76 (0.74-0.77) | 0.63 (0.60-0.65) |
| high risk | 0.86 (0.81-0.90) | 0.69 (0.62-0.75) | 0.57 (0.49-0.65) |
| **CMV** |  |  |  |
| low risk | 0.88 (0.85-0.91) | 0.78 (0.73-0.83) | 0.66 (0.59-0.73) |
| intermediate risk | 0.89 (0.88-0.90) | 0.76 (0.74-0.78) | 0.63 (0.61-0.66) |
| high risk | 0.87 (0.85-0.90) | 0.71 (0.68-0.75) | 0.57 (0.52-0.62) |
| **Recipient Diabetes** |  |  |  |
| No | 0.91 (0.90-0.92) | 0.77 (0.75-0.79) | 0.66 (0.64-0.69) |
| Yes | 0.87 (0.86-0.89) | 0.74 (0.72-0.76) | 0.58 (0.55-0.61) |
| **Recipient Peripheral Vascular Disease** |  |  |  |
| No | 0.90 (0.89-0.91) | 0.76 (0.75-0.78) | 0.63 (0.61-0.65) |
| Yes | 0.85 (0.82-0.88) | 0.69 (0.65-0.73) | 0.57 (0.51-0.62) |
| **cPRA (%)** |  |  |  |
| 0-84 | 0.89 (0.88-0.90) | 0.75 (0.74-0.77) | 0.62 (0.60-0.64) |
| 85-90 | 0.73 (0.51-0.96) | 0.64 (0.38-0.90) | 0.64 (0.38-0.90) |
| 90-94 | 1.00 (1.00-1.00) | 0.93 (0.79-1.06) | 0.93 (0.79-1.06) |
| ≥95 | 0.93 (0.87-0.98) | 0.71 (0.60-0.82) | 0.63 (0.50-0.76) |
| **Renal Graft Biopsy** |  |  |  |
| No | 0.88 (0.84-0.92) | 0.77 (0.70-0.84) | 0.67 (0.58-0.77) |
| Yes | 0.89 (0.88-0.90) | 0.75 (0.74-0.77) | 0.62 (0.60-0.64) |
| **Donor Proteinuria** |  |  |  |
| No | 0.89 (0.88-0.90) | 0.75 (0.73-0.77) | 0.62 (0.59-0.65) |
| Yes | 0.89 (0.88-0.90) | 0.76 (0.74-0.78) | 0.63 (0.60-0.66) |
| **Donor Terminal Serum Sodium (mEq/L)** | | |  |
| <135 | 0.88 (0.81-0.94) | 0.68 (0.57-0.78) | 0.55 (0.42-0.68) |
| 135-145 | 0.89 (0.87-0.91) | 0.74 (0.72-0.77) | 0.62 (0.58-0.66) |
| >145 | 0.89 (0.88-0.90) | 0.76 (0.74-0.78) | 0.63 (0.60-0.65) |
| **CDC High Risk Donor** |  |  |  |
| No | 0.89 (0.88-0.90) | 0.75 (0.73-0.76) | 0.61 (0.59-0.64) |
| Yes | 0.91 (0.89-0.94) | 0.80 (0.75-0.84) | 0.71 (0.64-0.78) |
| **Donation after cardiocirculatory arrest** |  |  |  |
| No | 0.89 (0.88-0.90) | 0.76 (0.74-0.77) | 0.62 (0.60-0.64) |
| Yes | 0.84 (0.80-0.88) | 0.71 (0.65-0.77) | 0.64 (0.56-0.73) |
| **Use of Vasopressors for Donor Hemodynamic Stability** | |  |  |
| No | 0.88 (0.86-0.89) | 0.75 (0.72-0.77) | 0.62 (0.59-0.65) |
| Yes | 0.90 (0.89-0.91) | 0.76 (0.74-0.78) | 0.63 (0.60-0.66) |
| **Percentage of glomerulosclerosis on renal biopsy** | | |  |
| 0-5 | 0.89 (0.88-0.91) | 0.76 (0.74-0.78) | 0.64 (0.61-0.66) |
| 6-10 | 0.88 (0.86-0.90) | 0.74 (0.71-0.77) | 0.60 (0.55-0.65) |
| 11-15 | 0.88 (0.85-0.91) | 0.77 (0.72-0.81) | 0.62 (0.55-0.69) |
| 16-20 | 0.89 (0.85-0.93) | 0.73 (0.66-0.80) | 0.56 (0.46-0.66) |
| >20 | 0.94 (0.90-0.98) | 0.79 (0.72-0.87) | 0.62 (0.51-0.73) |
| Indeterminate | 0.71 (0.38-1.05) | 0.43 (0.06-0.80) | 0.29 (-0.05-0.62) |
| **Donor Peak serum creatinine (mg/dL)** |  |  |  |
| <1 | 0.89 (0.87-0.91) | 0.77 (0.74-0.80) | 0.62 (0.58-0.67) |
| 1-1.5 | 0.89 (0.88-0.91) | 0.75 (0.73-0.77) | 0.63 (0.60-0.66) |
| 1.51-2 | 0.89 (0.87-0.91) | 0.77 (0.74-0.80) | 0.66 (0.61-0.71) |
| >2 | 0.87 (0.85-0.90) | 0.73 (0.69-0.77) | 0.55 (0.49-0.62) |
| **Time on dialysis (years)** |  |  |  |
| <3 | 0.91 (0.89-0.92) | 0.80 (0.78-0.82) | 0.68 (0.64-0.71) |
| 3-<5 | 0.89 (0.87-0.91) | 0.73 (0.70-0.76) | 0.60 (0.55-0.64) |
| >5 | 0.87 (0.85-0.89) | 0.72 (0.70-0.74) | 0.59 (0.55-0.62) |
| **Delayed Graft Function** |  |  |  |
| No | 0.93 (0.92-0.94) | 0.80 (0.78-0.82) | 0.66 (0.63-0.69) |
| Yes | 0.81 (0.80-0.83) | 0.66 (0.64-0.69) | 0.55 (0.51-0.59) |

**Table S7** Unadjusted hazard ratio for the risk of graft loss after renal transplantation with high-KDPI kidneys.

| **Characteristics** | **Unadjusted Hazard Ratio** | **P value** |
| --- | --- | --- |
| **Recipient Age** |  |  |
| >65 yrs | Reference [1] |  |
| <35 yrs | 1.29(0.58-2.88) | 0.539 |
| 35-49 yrs | 0.73(0.55-0.97) | 0.032 |
| 50-64 yrs | 0.95(0.85-1.07) | 0.416 |
| **Recipient Sex** |  |  |
| Female | Reference [1] |  |
| Male | 1.16(1.03-1.31) | 0.016 |
| **Recipient Race-ethnicity** |  |  |
| White-Caucasian | Reference [1] |  |
| Black-African American | 1.04(0.91-1.19) | 0.595 |
| Hispanic-LatiNO | 0.94(0.80-1.10) | 0.435 |
| Asian | 0.73(0.57-0.93) | 0.011 |
| Other | 0.74(0.41-1.34) | 0.321 |
| **Recipient Body Mass Index (BMI)** |  |  |
| 18.5-24.9 | Reference [1] |  |
| <18.5 | 1.05(0.69-1.60) | 0.806 |
| 25-29.9 | 1.02(0.88-1.19) | 0.773 |
| ≥ 30 | 1.12(0.96-1.30) | 0.142 |
| **Recipient History of diabetes** |  |  |
| NO | Reference [1] |  |
| YES | 1.30(1.15-1.46) | <.0001 |
| **Recipient History of peripheral vascular disease** |  |  |
| NO | Reference [1] |  |
| YES | 1.37(1.18-1.59) | <.0001 |
| **Etiology of Renal Failure** |  |  |
| Diabetic neprhopathy | Reference [1] |  |
| Hypertension | 0.77(0.67-0.89) | 0.000 |
| Glomerulonephritis | 0.77(0.63-0.94) | 0.010 |
| Other | 0.75(0.61-0.92) | 0.006 |
| **c-PRA (%)** |  |  |
| 0-84 | Reference [1] |  |
| 85-90 | 1.74(0.72-4.20) | 0.215 |
| 90-94 | 0.19(0.03-1.37) | 0.101 |
| ≥ 95 | 0.91(0.60-1.39) | 0.661 |
| **Karnofsky Functional Index, n. (%)** |  |  |
| <50 (poor) | Reference [1] |  |
| 50-70 (fair) | 0.66(0.47-0.91) | 0.011 |
| >70 (good) | 0.49(0.36-0.68) | <.0001 |
| **KDPI Group** |  |  |
| 86-90 | Reference [1] |  |
| 91-95 | 1.03(0.90-1.17) | 0.711 |
| >95 | 1.12(0.95-1.31) | 0.180 |
| **Dialysis duration, years** |  |  |
| 0-3 | Reference [1] |  |
| 3.1-5 | 1.35(1.15-1.57) | 0.000 |
| > 5 | 1.44(1.25-1.65) | <.0001 |
| **EBV serous status** |  |  |
| Intermediate risk | Reference [1] |  |
| Low risk | 0.35(0.05-2.49) | 0.295 |
| High risk | 1.29(1.03-1.62) | 0.025 |
| **CMV serous status** |  |  |
| Intermediate risk | Reference [1] |  |
| Low risk | 0.94(0.75-1.17) | 0.547 |
| High risk | 1.21(1.05-1.39) | 0.009 |
| **HLA mismatch** |  |  |
| 0-3 | Reference [1] |  |
| 4 | 1.02(0.83-1.25) | 0.874 |
| 5 | 1.16(0.96-1.40) | 0.127 |
| 6 | 1.23(1.01-1.50) | 0.042 |
| **Donor Age** |  |  |
| >65 yrs | Reference [1] |  |
| <35 yrs | 1.71(0.91-3.20) | 0.096 |
| 35-49 yrs | 0.85(0.60-1.20) | 0.358 |
| 50-64 yrs | 1.11(0.98-1.26) | 0.105 |
| **Donation after cardiocirculatory arrest** |  |  |
| NO | Reference [1] |  |
| YES | 1.26(1.01-1.58) | 0.039 |
| **Donor Proteinuria** |  |  |
| NO | Reference [1] |  |
| YES | 0.98(0.87-1.10) | 0.702 |
| **Donor Peak serum creatinine, mEq/L** |  |  |
| <1.0 | Reference [1] |  |
| 1.0-1.5 | 1.01(0.87-1.18) | 0.872 |
| 1.51-2.0 | 0.95(0.78-1.14) | 0.550 |
| > 2.0 | 1.19(0.98-1.45) | 0.072 |
| **Donor Terminal serum sodium (mEq/L)** |  |  |
| <135 | Reference [1] |  |
| 135-145 | 0.80(0.56-1.15) | 0.224 |
| >145 | 0.77(0.54-1.09) | 0.139 |
| **CDC high risk status** |  |  |
| NO | Reference [1] |  |
| YES | 0.75(0.61-0.93) | 0.010 |
| **Need of vasopressor for hemodynamic stability** |  |  |
| NO | Reference [1] |  |
| YES | 0.92(0.82-1.03) | 0.162 |
| **Biopsy performed** |  |  |
| NO | Reference [1] |  |
| YES | 1.05(0.79-1.39) | 0.753 |
| **Percentage of glomerulosclerosis identified in the surgical pathology specimen** | |  |
| 0-5% | Reference [1] |  |
| 6-10% | 1.08(0.93-1.24) | 0.316 |
| 11-15% | 1.06(0.86-1.31) | 0.563 |
| 16-20% | 1.14(0.88-1.49) | 0.321 |
| >20% | 0.96(0.70-1.32) | 0.807 |
| Immediate | 2.38(0.99-5.74) | 0.054 |
| **Kidneys put on perfusion pump** |  |  |
| NO | Reference [1] |  |
| YES | 1.01(0.90-1.14) | 0.811 |
| **Resistive index of kidneys while on perfusion pump** |  |  |
| ≤0.4 | Reference [1] |  |
| >0.4 | 1.45(1.02-2.05) | 0.037 |
| Missing | 1.01(0.90-1.14) | 0.864 |
| **Cold ischemia time, hours** |  |  |
| < 12 | Reference [1] |  |
| 12-17.9 | 1.12(0.92-1.37) | 0.243 |
| 18-23.9 | 1.21(1.00-1.46) | 0.054 |
| ≥ 24 | 1.20(0.99-1.45) | 0.065 |
| **Delayed Graft Function** |  |  |
| NO | Reference [1] |  |
| YES | 1.75(1.55-1.96) | <.0001 |

**Abbreviations:** BMI, body mass index; cPRA, calculated panel reactive antibody; KDPI, Kidney Donor Profile Index; EBV, Epstein–Barr virus; CMV, cytomegalovirus; HLA, human leukocyte antigen; CDC, Centers for Disease Control and Prevention.

**Table S8** Unadjusted hazard ratio for the risk of death-censored graft loss after renal transplantation with high-KDPI kidneys.

| **Characteristics** | **Unadjusted Hazard Ratio** | **P value** |
| --- | --- | --- |
| **Recipient Age, years** |  |  |
| >65 | Reference [1] |  |
| <35 | 3.87 (1.72-8.71) | 0.001 |
| 35-49 | 1.69 (1.20-2.37) | 0.002 |
| 50-64 | 1.30 (1.08-1.56) | 0.006 |
| **Recipient Sex** |  |  |
| Female | Reference [1] |  |
| Male | 1.22 (1.01-1.47) | 0.040 |
| **Recipient Race-ethnicity** |  |  |
| White-Caucasian | Reference [1] |  |
| Black-African American | 1.33 (1.09-1.63) | 0.005 |
| Hispanic-Latino | 0.81 (0.62-1.06) | 0.131 |
| Asian | 0.65 (0.43-0.97) | 0.036 |
| Other | 0.33 (0.08-1.31) | 0.114 |
| **Recipient Body Mass Index (BMI)** |  |  |
| 18.5-24.9 | Reference [1] |  |
| <18.5 | 0.31 (0.10-0.98) | 0.046 |
| 25-29.9 | 1.11 (0.88-1.40) | 0.379 |
| ≥ 30 | 1.25 (0.99-1.58) | 0.056 |
| **Recipient History of diabetes** |  |  |
| NO | Reference [1] |  |
| YES | 1.10 (0.93-1.32) | 0.275 |
| **Recipient History of peripheral vascular disease** |  |  |
| NO | Reference [1] |  |
| YES | 1.22 (0.96-1.54) | 0.098 |
| **Recipient Etiology of Renal Failure** |  |  |
| Diabetic nephropathy | Reference [1] |  |
| Hypertension | 0.82 (0.66-1.02) | 0.074 |
| Glomerulonephritis | 0.96 (0.72-1.28) | 0.783 |
| Other | 0.79 (0.58-1.08) | 0.139 |
| **cPRA (%)** |  |  |
| 0-84 | Reference [1] |  |
| 85-90 | 1.55 (0.39-6.21) | 0.538 |
| 90-94 | 0.45 (0.06-3.19) | 0.423 |
| ≥ 95 | 1.39 (0.82-2.37) | 0.222 |
| **Recipient Karnofsky Functional Index, n. (%)** |  |  |
| <50 (poor) | Reference [1] |  |
| 50-70 (fair) | 0.50 (0.32-0.77) | 0.002 |
| >70 (good) | 0.40 (0.26-0.62) | <.0001 |
| **KDPI Group** |  |  |
| 86-90 | Reference [1] |  |
| 91-95 | 1.16(0.96-1.41) | 0.125 |
| >95 | 1.07(0.84-1.37) | 0.582 |
| **Dialysis duration, years** |  |  |
| 0-3 | Reference [1] |  |
| 3.1-5 | 1.17(0.92-1.49) | 0.198 |
| > 5 | 1.48(1.21-1.82) | 0.000 |
| **Recipient EBV serous status** |  |  |
| Intermediate risk | Reference [1] |  |
| Low risk | 0.00(0.00--) | 0.961 |
| High risk | 1.42(1.02-1.98) | 0.039 |
| **Recipient CMV serous status** |  |  |
| Intermediate risk | Reference [1] |  |
| Low risk | 0.80(0.56-1.15) | 0.229 |
| High risk | 1.37(1.11-1.68) | 0.003 |
| **HLA mismatch** |  |  |
| 0-3 | Reference [1] |  |
| 4 | 1.12(0.82-1.51) | 0.478 |
| 5 | 1.13(0.85-1.50) | 0.410 |
| 6 | 1.10(0.81-1.50) | 0.530 |
| **Donor Age, years** |  |  |
| >65 | Reference [1] |  |
| <35 | 4.06(2.07-7.97) | <.0001 |
| 35-49 | 0.78(0.44-1.38) | 0.395 |
| 50-64 | 1.21(1.00-1.47) | 0.056 |
| **Donation After Cardiocirculatory Arrest** |  |  |
| NO | Reference [1] |  |
| YES | 1.33(0.96-1.83) | 0.082 |
| **Donor Proteinuria** |  |  |
| NO | Reference [1] |  |
| YES | 1.06(0.89-1.26) | 0.553 |
| **Donor Peak Serum Creatinine (mg/dL)** |  |  |
| <1.0 | Reference [1] |  |
| 1.0-1.5 | 0.91(0.72-1.14) | 0.401 |
| 1.51-2.0 | 0.90(0.68-1.19) | 0.466 |
| > 2.0 | 1.22(0.93-1.62) | 0.158 |
| **Donor Terminal Serum Sodium (mEq/L)** |  |  |
| <135 | Reference [1] |  |
| 135-145 | 0.77(0.45-1.30) | 0.327 |
| >145 | 0.73(0.43-1.22) | 0.227 |
| **Donor CDC High Risk Status** |  |  |
| NO | Reference [1] |  |
| YES | 0.72(0.52-1.01) | 0.054 |
| **Use of Vasopressor for Donor Hemodynamic Stability** |  |  |
| NO | Reference [1] |  |
| YES | 0.94(0.79-1.12) | 0.478 |
| **Renal Biopsy Performed** |  |  |
| NO | Reference [1] |  |
| YES | 0.82(0.56-1.20) | 0.310 |
| **Percentage of glomerulosclerosis identified in the surgical pathology specimen** | |  |
| 0-5% | Reference [1] |  |
| 6-10% | 1.10(0.88-1.37) | 0.392 |
| 11-15% | 1.04(0.75-1.44) | 0.830 |
| 16-20% | 1.26(0.85-1.86) | 0.247 |
| >20% | 1.28(0.83-1.98) | 0.268 |
| Indeterminate | 2.43(0.61-9.75) | 0.210 |
| **Kidneys put on perfusion pump** |  |  |
| NO | Reference [1] |  |
| YES | 1.01(0.85-1.21) | 0.879 |
| **Resistive index of kidneys while on perfusion pump** |  |  |
| ≤0.4 | Reference [1] |  |
| >0.4 | 1.35(0.79-2.32) | 0.278 |
| Missing | 1.03(0.86-1.23) | 0.775 |
| **Cold ischemia time, hours** |  |  |
| < 12 | Reference [1] |  |
| 12-17.9 | 1.03(0.77-1.38) | 0.831 |
| 18-23.9 | 1.10(0.83-1.45) | 0.506 |
| ≥ 24 | 1.00(0.76-1.34) | 0.976 |
| **Delayed Graft Function** |  |  |
| NO | Reference [1] |  |
| YES | 2.33(1.95-2.77) | <.0001 |

**Abbreviations:** BMI, body mass index; cPRA, calculated panel reactive antibody; KDPI, Kidney Donor Profile Index; EBV, Epstein–Barr virus; CMV, cytomegalovirus; HLA, human leukocyte antigen; CDC, Centers for Disease Control and Prevention.

**Supplementary Table S9. Sensitivity Analysis: Multivariable Logistic Regression Model for Delayed Graft Function Excluding Preemptively Transplanted Recipients**

This table presents results from a sensitivity analysis using a multivariable logistic regression model evaluating independent predictors of delayed graft function (DGF), defined as the need for dialysis within 7 days after transplantation, among recipients of kidneys with Kidney Donor Profile Index (KDPI) >85%. Analyses were restricted to recipients who underwent transplantation after initiation of dialysis, excluding those who were transplanted preemptively, to reduce potential outcome misclassification. Results are reported as regression coefficients, standard errors, adjusted odds ratios (aORs), 95% confidence intervals (CIs), and corresponding P values. Reference categories are indicated for categorical variables. All variables were selected a priori and entered simultaneously into the fully adjusted model.

| **Variables** | **Coeff** | **Standard Error** | **aOR** | **95% C.I.** | | **P-Value** |
| --- | --- | --- | --- | --- | --- | --- |
|  |  |  |  | **Lower** | **Upper** |  |
| ***Female Sex (Reference)*** | | | | | | **<.001** |
| Male Sex | 0.439 | 0.078 | **1.551** | 1.331 | 1.808 |  |
| ***Recipient Race*** | | | | | | **<.001** |
| White-Caucasian (Reference) |  |  |  |  |  |  |
| Black-African American | 0.432 | 0.096 | **1.540** | 1.277 | 1.858 | **<.001** |
| Hispanic-Latino | 0.613 | 0.144 | **1.847** | 1.394 | 2.447 | **<.001** |
| Asian | 0.236 | 0.311 | 1.266 | 0.688 | 2.329 | 0.449 |
| Other | 0.324 | 0.110 | **1.383** | 1.116 | 1.714 | **0.003** |
| ***BMI Category*** | | | | | | **<.001** |
| Normal Weight (Reference) |  |  |  |  |  |  |
| Overweight | 0.168 | 0.098 | 1.183 | 0.976 | 1.433 | 0.086 |
| Obese | 0.514 | 0.098 | **1.672** | 1.379 | 2.026 | **<.001** |
| Underweight | -0.233 | 0.407 | 0.792 | 0.357 | 1.759 | 0.567 |
| ***History of Peripheral Vascular Disease*** | 0.216 | 0.099 | **1.241** | 1.022 | 1.508 | **0.029** |
| ***Cause of renal failure*** | | | | | | 0.123 |
| Diabetic Nephropathy (Reference) |  |  |  |  |  |  |
| Hypertensive Nephropathy | -0.175 | 0.088 | **0.839** | 0.707 | 0.996 | **0.045** |
| Glomerulonephritis | -0.200 | 0.125 | 0.819 | 0.641 | 1.046 | 0.109 |
| Other | -0.175 | 0.130 | 0.839 | 0.650 | 1.083 | 0.178 |
| ***Functional Status*** | | | | | | 0.115 |
| Poor (Reference) |  |  |  |  |  |  |
| Fair | 0.085 | 0.247 | 1.089 | 0.671 | 1.766 | 0.731 |
| Good | -0.070 | 0.248 | 0.933 | 0.574 | 1.516 | 0.779 |
| ***Dialysis Duration*** | | | | | | **<.001** |
| ≤3 years (Reference) |  |  |  |  |  |  |
| 3.1-5 years | 0.253 | 0.096 | **1.287** | 1.066 | 1.555 | **0.009** |
| > 5 years | 0.607 | 0.091 | **1.836** | 1.534 | 2.196 | **<.001** |
| ***Donor Age Category*** | | | | | | 0.108 |
| ≥ 65 years (Reference) |  |  |  |  |  |  |
| < 35 years | -0.245 | 1.260 | 0.782 | 0.066 | 9.254 | 0.846 |
| 35-49.9 years | -0.222 | 0.200 | 0.801 | 0.541 | 1.186 | 0.268 |
| 50-64.9 years | -0.206 | 0.085 | **0.814** | 0.689 | 0.960 | **0.015** |
| ***Donor Meet CDC High-risk Category*** | -0.109 | 0.116 | 0.897 | 0.714 | 1.126 | 0.348 |
| ***Donor with Proteinuria*** | 0.160 | 0.073 | **1.174** | 1.017 | 1.356 | **0.029** |
| **Donor Peak Creatinine Category** | | | | | | **<.001** |
| <1.0 (Reference) |  |  |  |  |  |  |
| 1.0-1.5 | 0.137 | 0.104 | 1.147 | 0.935 | 1.406 | 0.188 |
| 1.51-2.0 | 0.442 | 0.117 | **1.556** | 1.236 | 1.958 | **<.001** |
| >2 | 0.482 | 0.125 | **1.619** | 1.267 | 2.068 | **<.001** |
| ***Use of Vasopressors for Donor Hemodynamic Stability*** | -0.340 | 0.073 | **0.712** | 0.616 | 0.822 | **<.001** |
| ***Percentage of Graft Glomerulosclerosis*** | | | | | | 0.197 |
| 0-5% (Reference) |  |  |  |  |  |  |
| 6-10% | 0.113 | 0.089 | 1.119 | 0.941 | 1.332 | 0.204 |
| 11-15% | 0.196 | 0.129 | 1.216 | 0.945 | 1.566 | 0.128 |
| 16-20% | -0.172 | 0.170 | 0.842 | 0.604 | 1.174 | 0.311 |
| > 20% | 0.012 | 0.197 | 1.012 | 0.688 | 1.488 | 0.951 |
| Indeterminate | 1.448 | 0.940 | 4.254 | 0.674 | 26.851 | 0.124 |
| ***Use of Pulsatile Organ Perfusion Technology*** | -0.533 | 0.075 | **0.587** | 0.506 | 0.680 | **<.001** |
| ***Duration of Cold Ischemia Time*** | | | | | | **<.001** |
| < 12 hours (Reference) |  |  |  |  |  |  |
| 12-17.9 hours | 0.075 | 0.121 | 1.078 | 0.851 | 1.366 | 0.532 |
| 18-23.9 hours | 0.344 | 0.119 | **1.411** | 1.118 | 1.781 | **0.004** |
| ≥ 24 hours | 0.388 | 0.120 | **1.475** | 1.165 | 1.867 | **0.001** |

**Legend:** Std. Error standard error; CI confidence interval; KDPI Kidney Donor Profile Index; EBV Epstein-Barr virus; CMV Cytomegalovirus; CDC Centers for Disease Control and Prevention.

**Table S10. Sensitivity Analysis: Multivariable Logistic Regression Model for Primary Graft Nonfunction Excluding Preemptively Transplanted Recipients.** This table presents results from a sensitivity analysis using a multivariable logistic regression model assessing independent predictors of primary graft nonfunction (PGNF), defined as irreversible allograft failure resulting in permanent dialysis dependence or graft nephrectomy within 90 days after transplantation, among recipients of kidneys with KDPI >85%. Analyses were limited to recipients who underwent transplantation after dialysis initiation, excluding preemptively transplanted patients, to ensure robust classification of early graft failure. Results are shown as regression coefficients, standard errors, adjusted odds ratios (aORs), 95% confidence intervals (CIs), and P values. Reference categories are specified for categorical variables, and all covariates were selected a priori based on clinical relevance.

| **Variable** | **Coeff** | **Standard Error** | **aOR** | **95% C.I.** | | **P Value** |
| --- | --- | --- | --- | --- | --- | --- |
|  |  |  |  | **Lower** | **Upper** |  |
| **Recipient Race** |  |  |  |  |  | 0.105 |
| White-Caucasian (Reference) |  |  |  |  |  |  |
| Black-African American | 0.210 | 0.229 | 1.233 | 0.787 | 1.933 | 0.360 |
| Hispanic-Latino | -0.393 | 0.438 | 0.675 | 0.286 | 1.591 | 0.369 |
| Asian | -0.612 | 1.028 | 0.543 | 0.072 | 4.066 | 0.552 |
| Other | -0.517 | 0.321 | 0.596 | 0.318 | 1.119 | 0.107 |
| **BMI Category** |  |  |  |  |  | 0.236 |
| Normal weight (Reference) |  |  |  |  |  |  |
| Overweight | 0.292 | 0.266 | 1.339 | 0.796 | 2.253 | 0.271 |
| Obese | 0.523 | 0.258 | **1.687** | 1.017 | 2.798 | **0.043** |
| Underweight | -17.455 | 5738.135 | 0.000 | 0.000 | . | 0.998 |
| **Dialysis Duration** |  |  |  |  |  | 0.149 |
| ≤3 years |  |  |  |  |  |  |
| 3.1-5 years | 0.031 | 0.254 | 1.031 | 0.626 | 1.698 | 0.904 |
| > 5 years | 0.395 | 0.230 | 1.485 | 0.946 | 2.331 | 0.086 |
| **CMV Serostatus** |  |  |  |  |  | 0.128 |
| Low risk (Reference) |  |  |  |  |  |  |
| Intermediate risk | -0.209 | 0.333 | 0.811 | 0.422 | 1.559 | 0.530 |
| High risk | 0.251 | 0.352 | 1.286 | 0.645 | 2.562 | 0.475 |
| **Donor Age Category** |  |  |  |  |  | **0.005** |
| > 65 years (Reference) |  |  |  |  |  |  |
| <35 years | 2.261 | 0.706 | **9.594** | 2.404 | 38.284 | **0.001** |
| 35-49.9 years | -0.799 | 0.620 | 0.450 | 0.134 | 1.516 | 0.197 |
| 50-64.9 years | -0.086 | 0.223 | 0.918 | 0.592 | 1.422 | 0.701 |
| **Donor Category** |  |  |  |  |  |  |
| Donation after brain death (Reference) |  |  |  |  |  |  |
| Donation after cardiocirculatory arrest | 0.555 | 0.298 | 1.741 | 0.970 | 3.126 | 0.063 |
| **Donor Proteinuria** |  |  |  |  |  |  |
| No proteinuria (Reference) |  |  |  |  |  |  |
| Presence of proteinuria | -0.305 | 0.191 | 0.737 | 0.507 | 1.072 | 0.110 |
| **Donor Peak Creatinine Category** |  |  |  |  |  | 0.020 |
| < 1.0 mg/dL (Reference) |  |  |  |  |  |  |
| 1.0-1.5 mg/dL | -0.024 | 0.269 | 0.977 | 0.577 | 1.654 | 0.930 |
| 1.51-2.0 mg/dL | 0.267 | 0.299 | 1.306 | 0.727 | 2.348 | 0.372 |
| >2.0 mg/dL | 0.718 | 0.298 | 2.049 | 1.144 | 3.673 | 0.016 |
| **Donor Terminal Serum Sodium** |  |  |  |  |  | 0.352 |
| 135-145 (Reference) |  |  |  |  |  |  |
| <135 mEq/L | -0.294 | 0.498 | 0.745 | 0.281 | 1.979 | 0.555 |
| >145 mEq/L | -0.522 | 0.487 | 0.593 | 0.229 | 1.540 | 0.283 |
| **Use of vasopressor for donor hemodynamic stability** |  |  |  |  |  |  |
| No vasopressor use (Reference) |  |  |  |  |  |  |
| Vasopressor use | -0.408 | 0.198 | **0.665** | 0.451 | 0.981 | **0.040** |
| **Resistive Index** |  |  |  |  |  | 0.242 |
| ≤0.4 (Reference) |  |  |  |  |  |  |
| >0.4 | 0.610 | 0.495 | 1.841 | 0.698 | 4.854 | 0.217 |

**Legend:** sHR Subdistribution hazard ratio; Std. Error standard error; CI confidence interval; KDPI Kidney Donor Profile Index; EBV Epstein-Barr virus; CMV Cytomegalovirus; CDC Centers for Disease Control and Prevention.

**Table S11. Sensitivity Analysis: Fine–Gray Competing-Risk Regression of Factors Associated With Graft Failure Among Recipients of High-KDPI Kidneys**

This table presents results from a sensitivity analysis using Fine–Gray competing-risk regression to evaluate factors associated with kidney graft failure among recipients of kidneys with Kidney Donor Profile Index (KDPI) >85%. The model estimates subdistribution hazard ratios (sHRs) for graft failure while treating death with a functioning graft as a competing event rather than a censoring event. Results are reported as regression coefficients, standard errors, subdistribution hazard ratios (sHRs), 95% confidence intervals (CIs), Z statistics, and corresponding P values. An sHR greater than 1 indicates a higher cumulative incidence of graft failure, whereas an sHR less than 1 indicates a lower cumulative incidence, after accounting for competing mortality. This analysis was performed to assess the robustness of findings from cause-specific Cox models.

| **Variable** | **Coefficient** | **sHR** | **Std. Error** | **Z** | **P Value** | **CI 2.5.** | **CI 97.5.** |
| --- | --- | --- | --- | --- | --- | --- | --- |
| **Recipient Age < 35** | **1.902** | **6.697** | **0.367** | **5.187** | **<.001** | **3.265** | **13.740** |
| Recipient Age 35-49 | 0.256 | 1.292 | 0.220 | 1.165 | 0.240 | 0.839 | 1.989 |
| Recipient Age 50-64 | 0.137 | 1.147 | 0.114 | 1.202 | 0.230 | 0.917 | 1.435 |
| Male Sex | 0.124 | 1.132 | 0.109 | 1.132 | 0.260 | 0.914 | 1.401 |
| Recipient Race: Black-African American | 0.084 | 1.088 | 0.135 | 0.621 | 0.530 | 0.834 | 1.418 |
| **Recipient Race: Hispanic-Latino** | **-0.635** | **0.530** | **0.248** | **-2.555** | **0.011** | **0.326** | **0.863** |
| Recipient Race: Other | -0.954 | 0.385 | 0.708 | -1.347 | 0.180 | 0.096 | 1.544 |
| **Recipient Race: Asian** | **-0.569** | **0.566** | **0.183** | **-3.106** | **0.002** | **0.395** | **0.811** |
| Recipient BMI: Underweight | 0.107 | 1.113 | 0.136 | 0.784 | 0.430 | 0.852 | 1.454 |
| Recipient BMI: Overweight | 0.111 | 1.117 | 0.138 | 0.802 | 0.420 | 0.852 | 1.465 |
| Recipient BMI: Obese | -1.449 | 0.235 | 0.974 | -1.488 | 0.140 | 0.035 | 1.583 |
| Cause of renal failure: Hypertension | -0.142 | 0.868 | 0.123 | -1.153 | 0.250 | 0.681 | 1.105 |
| Cause of renal failure: Glomerulonephritis | 0.153 | 1.166 | 0.159 | 0.966 | 0.330 | 0.854 | 1.592 |
| Cause of renal failure: Other | -0.039 | 0.961 | 0.184 | -0.214 | 0.830 | 0.670 | 1.380 |
| **Recipient functional status: Fair** | **-0.772** | **0.462** | **0.255** | **-3.022** | **0.003** | **0.280** | **0.762** |
| **Recipient functional status: Good** | **-0.911** | **0.402** | **0.254** | **-3.592** | **<.001** | **0.245** | **0.661** |
| KDPI 91-95% | 0.205 | 1.227 | 0.115 | 1.785 | 0.074 | 0.980 | 1.537 |
| KDPI >95% | 0.183 | 1.201 | 0.151 | 1.208 | 0.230 | 0.892 | 1.616 |
| Dialysis Duration: 3-5 years | 0.016 | 1.016 | 0.145 | 0.107 | 0.920 | 0.764 | 1.351 |
| Dialysis Duration: >5 years | 0.138 | 1.148 | 0.132 | 1.041 | 0.300 | 0.885 | 1.488 |
| **EBV: Intermediate Risk** | **7.495** | **1799.163** | **0.495** | **15.145** | **<.001** | **682.063** | **4745.878** |
| **EBV: High Risk** | **7.857** | **2582.966** | **0.521** | **15.089** | **<.001** | **930.917** | **7166.822** |
| **CMV: Intermediate Risk** | **0.454** | **1.574** | **0.226** | **2.004** | **0.045** | **1.010** | **2.454** |
| **CMV: High Risk** | **0.710** | **2.034** | **0.239** | **2.970** | **0.003** | **1.273** | **3.249** |
| **Donor Age: < 35 years** | **2.225** | **9.251** | **0.396** | **5.617** | **<.001** | **4.257** | **20.106** |
| Donor Age: 35-49 years | -0.339 | 0.712 | 0.330 | -1.030 | 0.300 | 0.373 | 1.359 |
| Donor Age: 50-64 years | 0.188 | 1.206 | 0.125 | 1.506 | 0.130 | 0.945 | 1.540 |
| Donor Peak Serum Creatinine: 1.0-1.5 (mg/dL) | -0.226 | 0.798 | 0.138 | -1.642 | 0.100 | 0.609 | 1.045 |
| Donor Peak Serum Creatinine: 1.51-2.0 (mg/dL) | -0.201 | 0.818 | 0.164 | -1.227 | 0.220 | 0.594 | 1.128 |
| Donor Peak Serum Creatinine: > 2.0 (mg/dL) | 0.075 | 1.078 | 0.165 | 0.454 | 0.650 | 0.780 | 1.489 |
| **CDC High Risk Donor** | **-0.394** | **0.674** | **0.199** | **-1.982** | **0.048** | **0.457** | **0.996** |
| Use of Pulsatile Organ Perfusion Technology | 0.168 | 1.182 | 0.107 | 1.560 | 0.120 | 0.958 | 1.460 |
| Terminal Serum Sodium: 135-145 (mEq/L) | -0.267 | 0.766 | 0.298 | -0.896 | 0.370 | 0.427 | 1.373 |
| Terminal Serum Sodium: > 145 (mEq/L) | -0.375 | 0.688 | 0.292 | -1.283 | 0.200 | 0.388 | 1.219 |
| Use of Vasopressors for Donor Hemodynamic Stability | -0.044 | 0.957 | 0.104 | -0.425 | 0.670 | 0.781 | 1.173 |
| Resistive Index: >0.4 | 0.222 | 1.248 | 0.358 | 0.619 | 0.540 | 0.618 | 2.520 |
| Cold Ischemia Time: 12-17.9 (hours) | 0.008 | 1.008 | 0.172 | 0.048 | 0.960 | 0.720 | 1.411 |
| Cold Ischemia Time 18-23.9 (hours) | 0.047 | 1.048 | 0.171 | 0.276 | 0.780 | 0.750 | 1.466 |
| Cold Ischemia Time: ≥24 (hours) | -0.014 | 0.986 | 0.176 | -0.078 | 0.940 | 0.699 | 1.392 |
| **Development of Delayed Graft Function** | **0.831** | **2.297** | **0.108** | **7.732** | **<.001** | **1.860** | **2.835** |

**Legend:** sHR Subdistribution hazard ratio; Std. Error standard error; CI confidence interval; KDPI Kidney Donor Profile Index; EBV Epstein-Barr virus; CMV Cytomegalovirus; CDC Centers for Disease Control and Prevention.
